# LtransHeteroGGM: local transfer learning for Gaussian graphical model-based heterogeneity analysis

**DOI:** 10.1093/bioinformatics/btag057

**Published:** 2026-02-04

**Authors:** Chengye Li, Hongwei Ma, Mingyang Ren

**Affiliations:** School of Mathematical Sciences, Shanghai Jiao Tong University, Shanghai 200240, China; Department of Critical Care Medicine, Xijing Hospital, Air Force Medical University (the Fourth Military Medical University), Shaanxi 710032, China; School of Mathematical Sciences, Shanghai Jiao Tong University, Shanghai 200240, China

## Abstract

**Motivation:**

Heterogeneity is a hallmark of both macroscopic complex diseases and microscopic single-cell distribution. Gaussian graphical models (GGMs)-based heterogeneity analysis highlights its important role in capturing the essential characteristics of biological regulatory networks, but faces instability with scarce samples from rare subgroups. Transfer learning offers promise by leveraging auxiliary data, yet existing approaches rely on unrealistic overall similarity between domains, requiring the same subgroup number and similar parameters. Numerous biological problems call for local similarities, where only some subgroups share statistical structures.

**Results:**

In this article, we propose LtransHeteroGGM, a novel local transfer learning framework for GGM-based heterogeneity analysis. It can achieve powerful subgroup-level local knowledge transfer between target and informative auxiliary domains, despite unknown subgroup structures and numbers, while mitigating the negative interference of non-informative domains. The effectiveness and robustness of the proposed approach are demonstrated through comprehensive numerical simulations and real-world T-cell heterogeneity analysis.

**Availability and implementation:**

The R implementation of LtransHeteroGGM is available at https://github.com/Ren-Mingyang/LtransHeteroGGM.

## 1 Introduction

Heterogeneity stands as a defining characteristic of complex diseases, including cancer, autoimmune disorders, and neurodegenerative conditions. Patients sharing a common diagnosis often exhibit substantial divergence in clinical presentation, progression trajectories, and therapeutic responses. This macroscopic variability finds its roots in microscopic diversity: single-cell technologies reveal that even within an individual patient, tissues comprise functionally distinct cellular subgroups characterized by unique transcriptional, metabolic, and signaling profiles. This intricate multi-scale heterogeneity presents a fundamental challenge to understanding disease mechanisms and developing effective, personalized therapies. Consequently, the precise dissection of heterogeneous structures is not merely a statistical exercise but a critical biological imperative for uncovering disease etiology and identifying actionable therapeutic targets. In statistical terminology, a data heterogeneous structure refers to the existence of latent distinct subgroups within a single domain, where the underlying distributions differ across these subgroups. For instance, the heterogeneous structure in single-cell sequencing data is often reflected in the statistically significantly different mean expression levels of biomarker genes across distinct cellular subgroups.

Traditional heterogeneity analysis has relied heavily on univariate or low-order distributional features, such as mean expression levels or variance of key biomarkers. While these approaches have yielded valuable insights, they fall short of capturing the higher-order dependencies that fundamentally define cellular states and functional units. Biological processes are orchestrated through complex molecular networks, where conditional dependencies, such as gene–gene interactions within regulatory networks, offer a more profound understanding of phenotypic divergence than marginal statistics alone. The feasibility of this paradigm is now established through advances in high-throughput omics technologies enabling network reconstruction, coupled with mature computational methods for network comparison. Its applicability extends to critical areas, including molecular disease subtyping based on network dysregulation patterns and uncovering mechanistic drivers of treatment response heterogeneity, making it a vital framework for moving beyond aggregate measures to decipher system-level biological variation. Gaussian graphical models (GGMs, Yuan and Lin 2007, Friedman *et al.* 2008) provide a rigorous statistical framework to model these conditional dependency structures, representing variables as nodes and their conditional independencies as edges in an undirected graph. For example, in single-cell RNA sequencing data (scRNA-seq), clustering cells based on GGM-inferred cell-type-specific interaction networks offers greater biological fidelity and resolution than methods relying solely on expression levels (Zhang *et al.* 2024). Thus, advancing beyond “means and variances” toward network-driven heterogeneity analysis represents a crucial step forward for precision biology. Under heterogeneity, when the subgrouping structure is known, Guo *et al.* (2011) developed a hierarchical penalty that targets removing common zero elements shared by subgroups. Danaher *et al.* (2014) adopted a single fusion penalty applied to pairwise differences between subgroups. A more challenging and more realistic scenario is where the subgrouping structure is unknown. Two representative works combine the Gaussian graphical mixture models (GGMMs) with the truncated fusion penalty applied to pairwise differences between subgroups (Gao *et al.* 2016) and the group penalty applied to elements across subgroups (Hao *et al.* 2018). More information can be referred to Tsai *et al.* (2022).

However, GGM-based heterogeneity analysis faces a significant practical limitation: the frequent scarcity of samples for biologically important but rare cellular subtypes or disease states. This data paucity leads to unstable network estimation and diminished statistical power. Transfer learning emerges as a promising strategy to address this challenge by leveraging information from auxiliary datasets, such as public repositories, related disease models, or different experimental batches, to enhance learning within a target domain of interest. Recently, Cai and Wei (2021) proposed some minimax and adaptive transfer learning-based classifiers. Li *et al.* (2022a) proposed the trans-lasso method under high-dimensional linear models with multiple auxiliary domains. This transfer learning framework is extended to high-dimensional generalized linear models (Tian and Feng 2023), federated learning (Li *et al.* 2023), and functional linear regression (Lin and Reimherr 2022). The unsupervised transfer learning remains largely underdeveloped (He *et al.* 2022, Li *et al.* 2022b). One may refer to Zhu *et al.* (2025) for more information. Moreover, existing approaches typically rely on a stringent assumption of overall similarity, requiring the overall distributional parameters between the target and auxiliary domains to be nearly identical, an assumption that rarely holds true in biological contexts where datasets originate from diverse cohorts, experimental conditions, or technological platforms.

**Figure 1:**
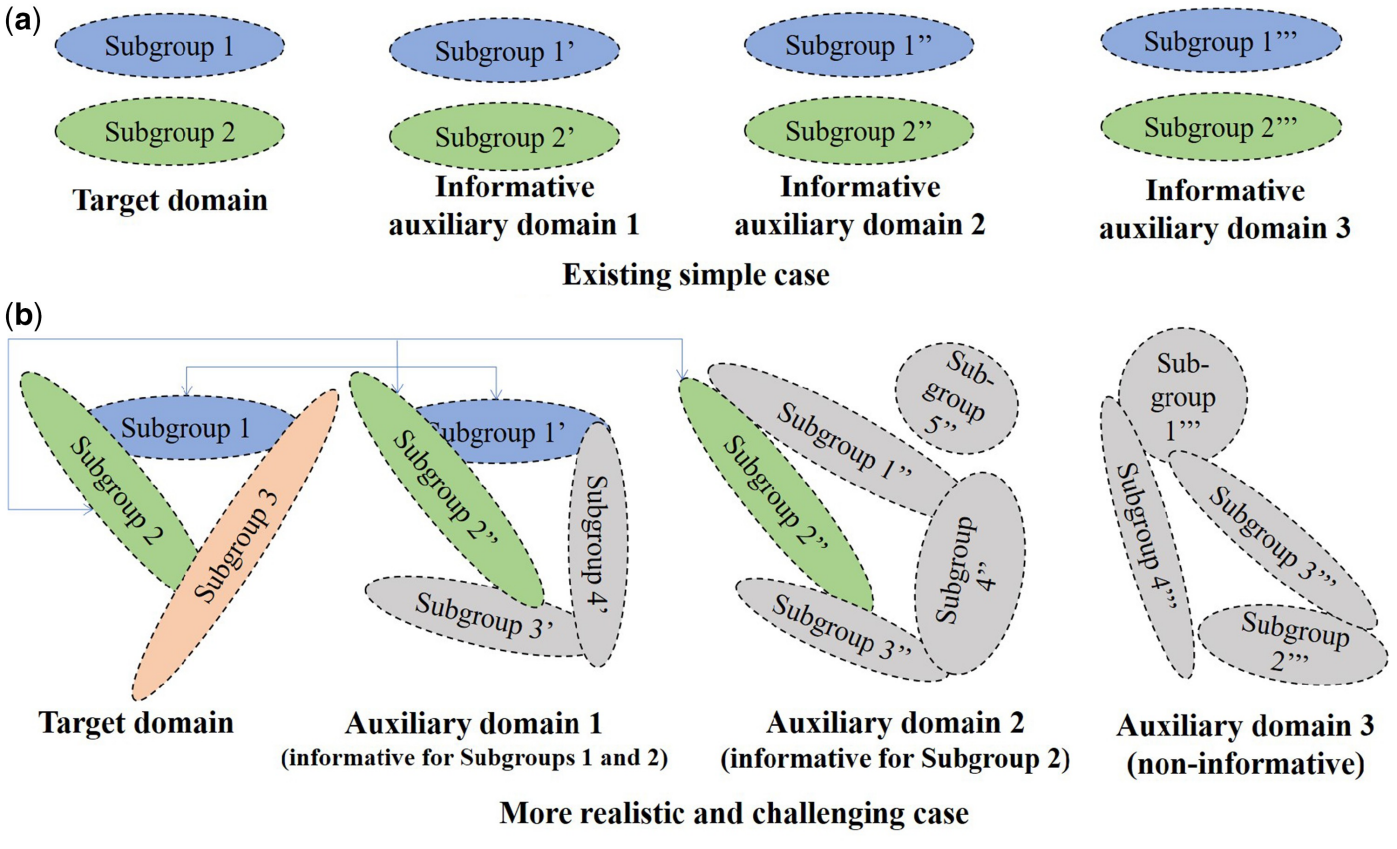
Toy GGMM examples for the existing case and the proposed more challenging case. The different shapes of subgroups roughly reflect differences in precision matrices.

Recent efforts to extend transfer learning to heterogeneous settings, such as Gaussian mixture graphical models, often remain constrained by this overall similarity paradigm (Tian *et al.* 2022, Wu *et al.* 2024). These methods typically demand that the target and auxiliary domains share an identical number of subgroups and exhibit closely aligned subgroup-specific parameters, as shown in Fig. 1a. This requirement is biologically unrealistic. Consider the analysis of CD4+ T cells in Sézary syndrome, an aggressive cutaneous T-cell lymphoma: target data might derive from the malignant CD4+ T cells from therapy-progressed patients, while auxiliary data could originate from cells with varying degrees of malignant transformation from early-stage patients. Although T cells exposed to different environmental stimuli may differentiate into subgroups with overlapping functions, i.e. their underlying subgroup architectures typically exhibit partial rather than complete correspondence, the overall similarity requirement for cells across domains is therefore biologically unrealistic. A more biologically plausible and flexible paradigm is local similarity, where the underlying parameters of the target and auxiliary data are similar only in some subgroup structures, as shown in Fig. 1b. More generally, auxiliary domains may contain numerous irrelevant subgroups, and some target subgroups might lack informative counterparts in the available auxiliary data. Although transfer learning approaches for internally heterogeneous data have emerged (Arash *et al.* 2025), the potential of networks to characterize the heterogeneity remains underexplored.

Performing GGM-based heterogeneity analysis under the local similarity paradigm introduces two core challenges: to define and quantify subgroup-level statistical similarity between heterogeneous domains rigorously, which is fundamental for identifying useful auxiliary information, and to prevent negative transfer, i.e. auxiliary data containing a preponderance of non-informative subgroups can overwhelm or mislead the learning process, leading to significant deviations from the true distribution of the target domain. To address these challenges, we propose LtransHeteroGGM, a novel framework specifically designed for local knowledge transfer in GGM-based heterogeneity analysis. Our approach follows a principled three-stage workflow. Initialization: employing efficient and initial GGM estimation for both target and auxiliary domains without requiring prior knowledge of subgroup numbers. Adaptive aggregation: prescreening auxiliary subgroups and assigning data-driven weights based on preliminary similarity assessments to mitigate the influence of irrelevant sources. Local transfer: performing knowledge transfer individually for each target subgroup, selectively leveraging information only from its most similar auxiliary counterparts, even if these are sparse or distributed across multiple auxiliary domains. This subgroup-specific local transfer maximizes relevant information gain while minimizing the risk of negative transfer from unrelated data. Through comprehensive simulations and analysis of real scRNA-seq from Sézary syndrome CD4+ T cells, we demonstrate that LtransHeteroGGM significantly enhances the accuracy of the target network, especially under limited sample sizes, and exhibits strong robustness against large numbers of non-informative auxiliary subgroups. The primary contributions of this work are three-fold:

- We propose LtransHeteroGGM, specifically designed for heterogeneous GGMs, providing a flexible solution for multi-source unsupervised data, and incorporating efficient algorithms for each stage.
- We explicitly address the challenging yet common scenario where only a few subgroups of auxiliary subgroups are relevant for any given target subgroup, with the completely unknown subgroup numbers and structures in all domains. We introduce a tailored adaptive weighting mechanism and local transfer strategy, providing robust solutions against negative transfer. Rigorous numerical experiments furnish compelling evidence of the method’s superiority and flexibility in complex biological data settings.
- We validate the biological utility and practical impact of LtransHeteroGGM through its application to complex disease data. The framework successfully enables cross-dataset knowledge transfer, such as different disease stages or cell contexts, in Sézary syndrome, leading to the identification of novel and significant hub genes in networks, thereby offering new insights into disease heterogeneity.

## 2 Materials and methods

We first present essential notations. Denote $∥u{∥}_{q}$ as the $l_{q}$-norm of a vector u, for q⩾0. For a matrix $A={\left(A_{ij}\right)}_{1⩽i,j⩽p}$, let $A_{j}$ be its *j*th column, $∥A{∥}_{q,\infty }=\max_{1⩽j⩽p}∥A_{j}{∥}_{q}$, $∥A{∥}_{1}=\sum_{j=1}^{p}∥A_{j}{∥}_{1}$, and $∥A{∥}_{1,\text{off}}=\sum_{1⩽i\neq j⩽p}|A_{ij}|$. [K]={1,…,K} be the *K*-set for any positive integer *K*. Moreover, preliminary concepts related to GGMMs are provided in Section A, available as supplementary data at *Bioinformatics* online.

### 2.1 Data and model setup

Considering transfer learning data setting, suppose that the target samples ${\left\{x_{i}\in R^{p}\right\}}_{i=1}^{n}$ and the auxiliary samples from *K* domains ${\left\{x_{i}^{\left(k\right)}\in R^{p}\right\}}_{i=1}^{n_{k}}$ for k=1,…,K are available, in which ${\left\{x_{i}\right\}}_{i=1}^{n}$ satisfy the GGMM $\sum_{l=1}^{L}\pi_{l}^{*}f_{l}\left(x;\mu_{l}^{*},{\left(\Theta_{l}^{*}\right)}^{-1}\right)$ consisting of *L* subgroups with $f_{l}$ defined by (S.1), ${\left\{x_{i}^{\left(k\right)}\right\}}_{i=1}^{n_{k}}$ satisfy the GGMM $\sum_{l=1}^{L^{\left(k\right)}}\pi_{l}^{(k)*}f_{l}(x;\mu_{l}^{(k)*},{\left(\Theta_{l}^{(k)*}\right)}^{-1})$ consisting of $L^{\left(k\right)}$ subgroups, and the auxiliary sample size $n_{k}\gg n$. Heterogeneity manifests at the sample level, where samples from different subgroups follow potentially distinct underlying distributions. It is assumed here that different domains share the same feature space; i.e. samples from all domains possess the same *p*-dimensional features. Note that there may not exist a single auxiliary domain with parameters ${\left\{\mu_{l}^{(k)*},\Theta_{l}^{(k)*}\right\}}_{l=1}^{L^{\left(k\right)}}$ that are similar to ${\left\{\mu_{l}^{*},\Theta_{l}^{*}\right\}}_{l=1}^{L}$ in the target domain, even *L* and ${\left\{L^{\left(k\right)}\right\}}_{k=1}^{K}$ can be unequal. Such local similarity is more flexible and realistic for heterogeneous GGMMs, which goes far beyond the overall similarity (Tian *et al.* 2022, Wu *et al.* 2024) and poses new challenges. Figure 1 provides an illustration of the difference between the existing literature and the proposed method.

It is clear that for any subgroup *l* of the target domain, we only expect that there exist some subgroups of some auxiliary domains, such that $A_{l}^{\Theta }=\{(l^{\prime },k):∥\Delta_{ll^{\prime }}^{(k)*}{∥}_{1,\infty }+∥{\left(\Delta_{ll^{\prime }}^{(k)*}\right)}^{⊤}{∥}_{1,\infty }⩽h_{\Theta }\}$ and $A_{l}^{\mu }=\{(l^{\prime },k):∥\delta_{ll^{\prime }}^{(k)*}{∥}_{1}⩽h_{\mu }\}$ are both non-empty for some small $h_{\Theta },\;h_{\mu }>0$, where $\Delta_{ll^{\prime }}^{(k)*}=\Theta_{l}^{*}\Sigma_{l^{\prime }}^{(k)*}-I_{p}$ and $\delta_{ll^{\prime }}^{(k)*}=\mu_{l}^{*}-\mu_{l^{\prime }}^{(k)*}$ measure the difference between the precision matrices and mean vectors of the *l*th target subgroup and the $l^{\prime }$th auxiliary subgroup in the *k*th domain, respectively. More detailed discussions on $h_{\Theta }$ are provided in Section B.3, available as supplementary data at *Bioinformatics* online. Note that the subgroup numbers *L* and $L^{\left(k\right)}$ as well as the subgroup memberships are all unknown, not to mention that one needs to accurately detect the most informative auxiliary domain for each subgroup in the target domain.

### 2.2 LtransHeteroGGM

To deal with the aforementioned challenging scenario, we propose a multi-step transfer learning framework, LtransHeteroGGM, summarized in Algorithm 1. In brief, the local knowledge transfer strategy improves the estimation of model parameters for the target subgroup by prescreening potentially informative auxiliary subgroups, adaptively aggregating them based on their parametric similarity to the target subgroup, and transferring their well-estimated model parameters.

*Step 1. Initialization.* We can obtain initial estimates in the target and auxiliary domains, respectively, under unknown numbers of subgroups using the HeteroGGM approach (Ren *et al.* 2022). It is estimated that there are $\hat{L}$ subgroups in the target domain with estimated mean vectors and precision matrices denoted as ${\left\{{\hat{\mu }}_{l}^{\left(0\right)},{\hat{\Theta }}_{l}^{\left(0\right)},{\hat{\pi }}_{l}^{\left(0\right)}\right\}}_{l=1}^{\hat{L}}$ and ${\hat{L}}^{\left(k\right)}$ subgroups in the *k*th auxiliary domain with ${\left\{{\hat{\mu }}_{l}^{\left(k\right)},{\hat{\Theta }}_{l}^{\left(k\right)},{\hat{\pi }}_{l}^{\left(k\right)}\right\}}_{l=1}^{{\hat{L}}^{\left(k\right)}}$, for k=1,…,K. For the initialization step, it is necessary to apply the penalized EM algorithm to all domains separately, and the $\Theta_{l}^{\left(0\right)}$ update in its *M*-step can correspond to a joint weighted graphical lasso algorithm (Danaher *et al.* 2014). We provide the detailed E-step and M-step as well as the stopping criteria in Section C, available as supplementary data at *Bioinformatics* online. Then, compute the pseudo sample covariance matrix of the *l*th subgroup in *k*th auxiliary domain, ${\tilde{\Sigma }}_{l}^{\left(k\right)}=\frac{\sum_{i=1}^{n_{k}}{\hat{\gamma }}_{il}^{\left(k\right)}(x_{i}^{\left(k\right)}-{\hat{\mu }}_{l}^{\left(k\right)}){\left(x_{i}^{\left(k\right)}-{\hat{\mu }}_{l}^{\left(k\right)}\right)}^{⊤}}{\sum_{i=1}^{n_{k}}{\hat{\gamma }}_{il}^{\left(k\right)}},$ where ${\hat{\gamma }}_{il}^{\left(k\right)}=\frac{{\hat{\pi }}_{l}^{\left(k\right)}f_{l}(x_{i}^{\left(k\right)};{\hat{\mu }}_{l}^{\left(k\right)},{\left[{\hat{\Theta }}_{l}^{\left(k\right)}\right]}^{-1})}{\sum_{l=1}^{{\hat{L}}^{\left(k\right)}}{\hat{\pi }}_{l}^{\left(k\right)}f_{l}(x_{i}^{\left(k\right)};{\hat{\mu }}_{l}^{\left(k\right)},{\left[{\hat{\Theta }}_{l}^{\left(k\right)}\right]}^{-1})}$, and define $n_{kl}=\sum_{i=1}^{n_{k}}{\hat{\gamma }}_{il}^{\left(k\right)}$ as the estimated pseudo sample size of the *l*th subgroup in *k*th auxiliary domain.

*Step 2. Prescreening and adaptive weighting.* For $l=1,\ldots ,\hat{L}$ of the target domain, calculate the divergence matrix between it and all auxiliary subgroups, ${\hat{\Delta }}_{ll^{\prime }}^{\left(k\right)}={\hat{\Theta }}_{l}^{\left(0\right)}{\tilde{\Sigma }}_{l^{\prime }}^{\left(k\right)}-I_{p}$, for k∈[K], $l^{\prime }\in [{\hat{L}}^{\left(k\right)}]$, and reserve the auxiliary subgroups such that $(l^{\prime },k)\in {\hat{A}}_{l}^{\Theta }$, where


(1) $${\hat{A}}_{l}^{\Theta }=\{(l^{\prime },k):∥{\hat{\Delta }}_{ll^{\prime }}^{\left(k\right)}{∥}_{1,\infty }+∥{\left({\hat{\Delta }}_{ll^{\prime }}^{\left(k\right)}\right)}^{⊤}{∥}_{1,\infty }⩽c\hat{s}\sqrt{\frac{\;\text{log}\;p}{n_{0l}}}\},$$

with $\hat{s}=∥{\hat{\Theta }}_{l}^{\left(0\right)}{∥}_{0,\infty }$, $n_{0l}=\sum_{i=1}^{n}{\hat{\gamma }}_{il}$, ${\hat{\gamma }}_{il}=\frac{{\hat{\pi }}_{l}^{\left(0\right)}f_{l}(x_{i};{\hat{\mu }}_{l}^{\left(0\right)},{\left[{\hat{\Theta }}_{l}^{\left(0\right)}\right]}^{-1})}{\sum_{l=1}^{{\hat{L}}^{\left(k\right)}}{\hat{\pi }}_{l}^{\left(0\right)}f_{l}(x_{i};{\hat{\mu }}_{l}^{\left(0\right)},{\left[{\hat{\Theta }}_{l}^{\left(0\right)}\right]}^{-1})}$, and a given positive number *c*. ${\hat{A}}_{l}^{\Theta }$ can be considered as a rough estimate of the informative auxiliary subgroup set for the *l*th target subgroup.

Then, the pseudo sample covariance matrices of the retained auxiliary subgroups are weighted according to their similarity with the target one and sample size. Particularly, we set ${\hat{\Sigma }}_{l}^{A}=\sum_{(l^{\prime },k)\in {\hat{A}}_{l}^{\Theta }}\alpha_{kl^{\prime }}{\tilde{\Sigma }}_{l^{\prime }}^{\left(k\right)}$, with


(2) $$\alpha_{kl^{\prime }}=\frac{n_{kl^{\prime }}/∥{\hat{\Delta }}_{ll^{\prime }}^{\left(k\right)}{∥}_{1,\infty }}{\sum_{(l^{\prime },k)\in {\hat{A}}_{l}^{\Theta }}\left(n_{kl^{\prime }}/∥{\hat{\Delta }}_{ll^{\prime }}^{\left(k\right)}{∥}_{1,\infty }\right)}.$$

Evidently, among auxiliary subgroups of comparable size, greater weighting is assigned to those exhibiting smaller deviations from the target subgroup, while weights for subgroups with pronounced differences may diminish to near-zero values to adaptively counteract negative transfer. A more in-depth discussion is provided in Section B.2, available as supplementary data at *Bioinformatics* online.

*Step 3. Local transfer.* In this step, the auxiliary information transfer is locally implemented on each target subgroup. Specifically, an adaptive thresholding on the divergence matrix between the target precision matrix and adaptively weighted pseudo sample covariance matrices, ${\hat{\Theta }}_{l}^{\left(0\right)}{\hat{\Sigma }}_{l}^{A}-I_{p}$, is first conducted by ${\hat{\Delta }}_{l}=\underset{\Delta }{\text{argmin}}\;(\frac{1}{2}\text{tr}\{\Delta^{⊤}\Delta \}-\text{tr}\{{\left({\hat{\Theta }}_{l}^{\left(0\right)}{\hat{\Sigma }}_{l}^{A}-I_{p}\right)}^{⊤}\Delta \}+\lambda_{1l}∥\Delta {∥}_{1})$, and then the estimator ${\left\{{\hat{\Theta }}_{l}\right\}}_{l=1}^{\hat{L}}$ can be obtained as


(3) $${\hat{\Theta }}_{l}=\underset{\Theta_{l}}{\text{argmin}}\;(\frac{1}{2}\text{tr}\{\Theta_{l}^{⊤}{\hat{\Sigma }}_{l}^{A}\Theta_{l}\}-\text{tr}\{({\hat{\Delta }}_{l}^{⊤}+I_{p})\Theta_{l}\}+\lambda_{2l}∥\Theta_{l}{∥}_{1,\text{off}}),$$

for $l=1,\ldots ,\hat{L}$. Step 3 involves optimizing two objective functions, and their detailed algorithm is provided in Section B, available as supplementary data at *Bioinformatics* online. As for the tuning parameter selection, we set $\lambda_{1l}=2∥{\hat{\Theta }}_{l}^{\left(0\right)}{∥}_{1,\infty }\sqrt{\frac{\;\text{log}\;p}{n}}$, following Li *et al.* (2022b). For $\lambda_{2l}$, it is suggested to be determined via minimizing a BIC-type criterion,


(4) $$\frac{1}{2}\text{tr}\{{\hat{\Theta }}_{l}^{⊤}{\hat{\Sigma }}_{l}^{\left(k\right)}{\hat{\Theta }}_{l}\}-\text{tr}\{({\hat{\Delta }}_{l}^{⊤}+I_{p}){\hat{\Theta }}_{l}\}+\frac{\;\text{log}\;n}{n}∥{\hat{\Theta }}_{l}{∥}_{0}.$$

Algorithm 1. Summary for LtransHeteroGGM

**Input**: The target samples ${\left\{x_{i}\in R^{p}\right\}}_{i=1}^{n}$ and the auxiliary samples from *K* domains ${\left\{x_{i}^{\left(k\right)}\in R^{p}\right\}}_{i=1}^{n_{k}}$ for k=1,…,K.

**Step 1**. Initialization for all domains via HeteroGGM.

**Step 2**. Adaptive auxiliary domain aggregation via (1)–(2).

**Step 3**. Local transfer for each target subgroup via (3) and select tuning parameters via (4).

The main concern in GGMs is the precision matrix; nevertheless, a similar procedure can be constructed if we also want to improve the estimation of the mean, which is provided in Section B.4, available as supplementary data at *Bioinformatics* online.

**Rationale**. In Step 1, the HeteroGGM method is employed to initialize the procedure, yielding initial estimates of the subgroup parameters as well as the number of subgroups within both the auxiliary domains and the target domain. These initial estimates are subsequently utilized to construct adaptive weights based on the discrepancy between the initial auxiliary and target subgroup parameters. Step 2 involves a prescreening step that constructs an empirically informative set of auxiliary subgroups ${\hat{A}}_{l}^{\Theta }=\{(l^{\prime },k):∥{\hat{\Delta }}_{ll^{\prime }}^{\left(k\right)}{∥}_{1,\infty }+∥{\left({\hat{\Delta }}_{ll^{\prime }}^{\left(k\right)}\right)}^{⊤}{∥}_{1,\infty }⩽c\hat{s}\sqrt{\frac{\;\text{log}\;p}{n_{0l}}}\}$ for each target subgroup *l*. This set selectively includes only those auxiliary domains where the initial estimated parameter differences fall below a specified threshold $c\hat{s}\sqrt{\frac{\;\text{log}\;p}{n_{0l}}}$. The threshold is intrinsically defined as the estimation error order derived solely from the target domain using a common single-task graphical model. The selection of this error order as the threshold is statistically justified, as auxiliary subgroups exhibiting parameter differences substantially exceeding this order are generally considered non-informative (Li *et al.* 2022b). This prescreening approach, utilizing a hard threshold to detect potentially informative auxiliary subgroups, shares the same spirit with established methodologies (Tian and Feng 2023). In our numerical implementation, we set c=5. The construction of adaptive weights in Step 2 serves as a dual safeguard against negative transfer. Even if auxiliary subgroups with significant deviations are inadvertently included in the prescreening, the adaptive weighting scheme is designed to minimize their influence as much as possible. Note that the prescreening and adaptive weighting steps are more applicable to GGM-based heterogeneity analysis than the simple model selection step between estimators based on all auxiliary domains and based on the target domain only (Li *et al.* 2022b) because the proposed step can still detect and utilize informative auxiliary subgroups to borrow this information even with the interference of non-informative ones, but the model selection may force the final estimator to become the initial estimate based on only the target subgroup as long as there are individual non-informative auxiliary subgroups with significant impact, which is very common due to strong heterogeneity across multiple subgroups from different auxiliary domains, so that information from another part of informative auxiliary subgroups may be offset. In this sense, the model selection can only ensure that their method does not deteriorate, but cannot make full use of informative auxiliary subgroups. In Step 3, ${\hat{\Delta }}_{l}$ can be considered as an adaptive thresholding of a naive estimate, ${\hat{\Theta }}_{l}^{\left(0\right)}{\hat{\Sigma }}_{l}^{A}-I_{p}$. If the difference between the target and weighted auxiliary subgroups is small enough, some elements of ${\hat{\Delta }}_{l}$ can shrink to zero with appropriate $\lambda_{1}$. The thresholding can improve the estimation of ${\hat{\Delta }}_{l}$ with the help of the auxiliary samples. Correspondingly, $\Theta_{l}$ can also be better estimated via ${\hat{\Theta }}_{l}$ by leveraging only the auxiliary samples in (3).

Although HeteroGGM shares similarities with the proposed LtransHeteroGGM in heterogeneous modeling, it serves merely as an initialization for LtransHeteroGGM. The data settings and methodologies considered in LtransHeteroGGM, including the multi-source heterogeneous data setting with local similarity, adaptive truncation and integration of auxiliary domains, local transfer methods, and others, far exceed the scope of HeteroGGM. A detailed comparison is provided in Table S1, available as supplementary data at *Bioinformatics* online.

Although the proposed method exhibits a moderate dependence on initialization, especially the correct number of subgroups, the safeguard against negative transfer inherent in the proposed framework ensures that initial bias is not amplified by the local transfer mechanism, even under poor initialization. This safety arises because significant distributional shifts in poorly initialized target subgroups (including the wrong subgroup number) relative to the true distribution typically result in the absence of closely matching auxiliary subgroups. Consequently, during the prescreening step of the framework, the informative auxiliary set can be identified as an empty set, leading to the immediate termination of information transfer. This mechanism effectively prevents the amplification of initial estimation errors.

## 3 Results

Simulation study is first conducted, in which we consider a three-subgroup (L=3) target domain with the number of variables p=100 and the sample size $n_{0}=200$ across all subgroups. Different subgroups follow Gaussian distributions with different mean vectors and precision matrices. Here, we consider the tridiagonal and block power-law network structures, with details in Section D.1, available as supplementary data at *Bioinformatics* online.

### 3.1 Setting of auxiliary domains

For auxiliary domains, we consider two different simulation examples. The objective of Example 1 is to demonstrate that the performance of the proposed approach improves with an increase in the number of informative auxiliary domains with overall similarity, while effectively filtering out interference from non-informative auxiliary domains. The objective of Example 2 is to demonstrate that the proposed method achieves robust local transfer in challenging situations where the informative auxiliary domain only has local similarity.

Example 1.(*Overall similarity*) We fix K=5 and vary the number of informative auxiliary domains card(A)  ∈{0,1,…,K}. All auxiliary domains have the same subgroup number L=3 as that of the target domain, and the sample size of all auxiliary subgroups is $3n_{0}$. The generation method of informative auxiliary domains is shown in Section D.1, available as supplementary data at *Bioinformatics* online.

Example 2. (*Local similarity*) We fix K=6 and the number of subgroups for *K* auxiliary domains are {2,3,4,3,3,3} respectively. The first three auxiliary domains are informative with local similarity, specifically, the *k*th subgroup of the *k*th informative auxiliary domain has similar parameters to the *k*th subgroup of the target domain, for k=1,2,3. Other auxiliary subgroups (whether in the first three informative or the last three non-informative auxiliary domains) are not similar to any target subgroup. The specific generation method of parameters and the sample size in auxiliary subgroups are similar to Example 1.

### 3.2 Competing methods

We compare our method with both some popular single-domain learning and recent transfer learning methods for GGMMs as follows. (i) A two-step approach denoted by “JGL.” *K*-means clustering combined with the gap statistic is first used to obtain the number and structures of subgroups, and then the joint graphical lasso (Danaher *et al.* 2014) is used to estimate the precision matrices. (ii) The SCAN method (Hao *et al.* 2018) with the correct subgroup number L=3. (iii) The SCAN method with the estimated number of subgroups in (i), denoted by “SCAN.v.” (iv) The HeteroGGM approach (Ren *et al.* 2022) without requiring a known subgroup number. (v) The transfer learning method for two-subgroup GGMMs (Tian *et al.* 2022), denoted by “tlgmm.” Admittedly, for the novel setting considered in this paper, scarcely any suitable existing methods are available for direct comparison. Consequently, we primarily compare against several methods based on a single dataset. The only transfer learning method suitable for comparison is tlgmm, which is an overall transfer limited by its strong assumptions of low dimensionality and bivariate subgroups with identical covariance. To ensure a fair comparison with tlgmm, we conducted further numerical analysis under scenarios compatible with its assumptions; these additional results are provided in Section E.2, available as supplementary data at *Bioinformatics* online.

### 3.3 Performance metrics

The performances of the competing methods are measured by a number of metrics: (i) mean and SD of *L*, (ii) percentage of $\hat{L}$ equal to *L*, denoted by “Per,” (iii) subgrouping error defined by $\text{CE}(\hat{\varphi },\varphi ):={\left(\begin{array}{c} n\\ 2 \end{array}\right)}^{-1}|\{(i,j):I\left(\hat{\varphi }\left(x_{i}\right)=\hat{\varphi }\left(x_{j}\right)\right)\neq I\left(\varphi \left(x_{i}\right)=\varphi \left(x_{j}\right)\right);i<j\}|$, where $\hat{\varphi }$ and φ are the estimated and true subgrouping memberships, respectively, (iv) average mean squared error (MSE) for mean vectors and precision matrices across all subgroups, and (v) average true and false positive rates (TPR and FPR) for the off-diagonal elements of the precision matrices across all subgroups. Note that a good estimation is implied with small values of CE, MSE, and FPR, but large values of Per and TPR.

### 3.4 Comparison conclusion

**Figure 2:**
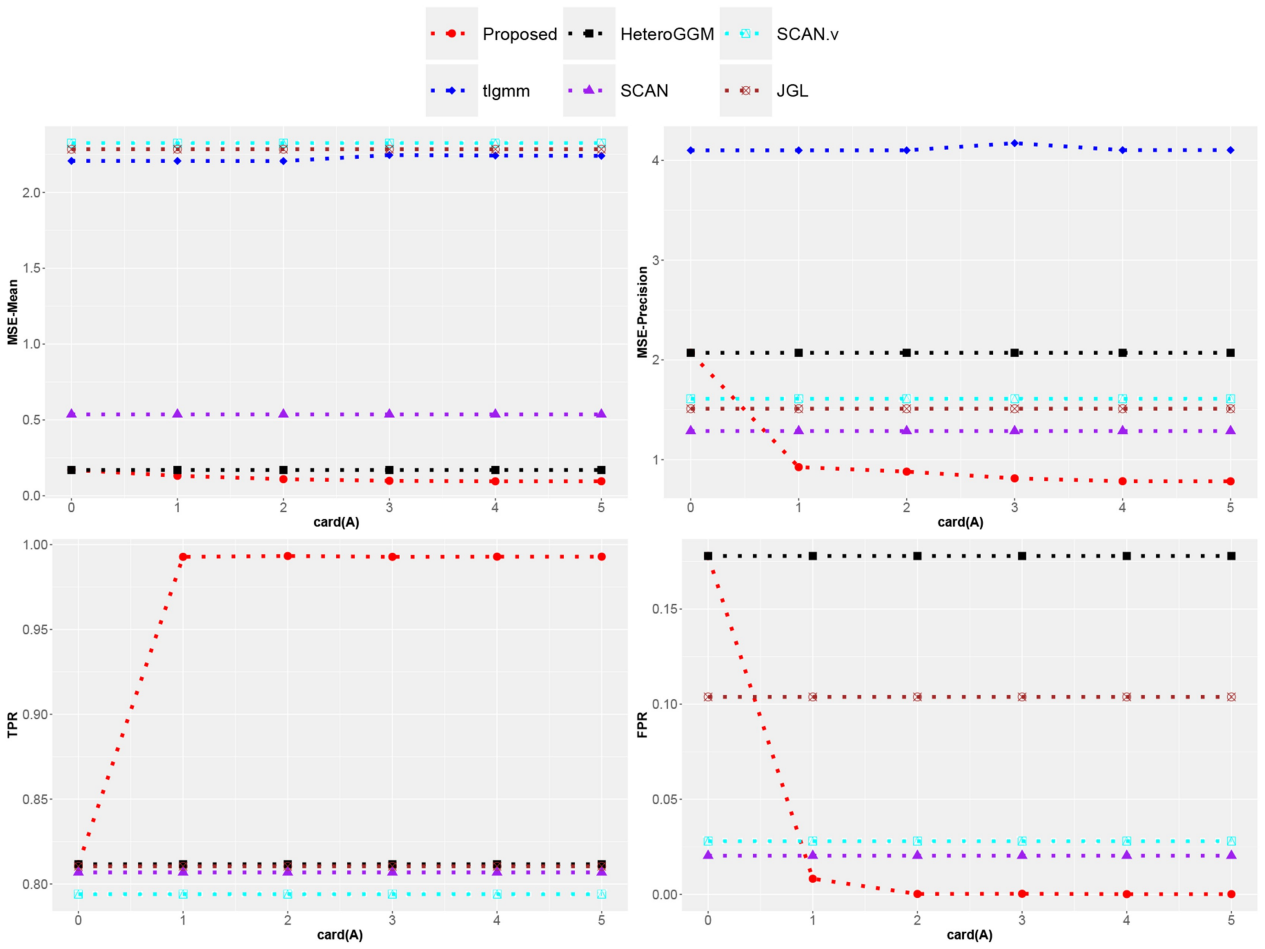
Averaged metrics of estimation errors over 100 replications for Example 1 with tridiagonal networks.

The averaged metrics over 100 independent replications under Examples 1 and 2 are summarized in Fig. 2 and Fig. S1, available as supplementary data at *Bioinformatics* online, and Table 1, whereas more numerical results are provided in Tables S1 and S2, available as supplementary data at *Bioinformatics* online. Observations made are very similar under different settings. For example, in Example 1, when card(A)=0, simulating the scenario where the target subgroup is highly specific without any informative auxiliary domain, there is little difference in the performance of the proposed method and the single-domain methods under different network settings, indicating that the proposed method would not reduce the analysis performance of the highly specific target domain, thanks to the safeguard on potential negative transfer. But once there is an informative auxiliary domain with overall similarity, the proposed transfer learning method will outperform other competitors significantly, even if there is interference from some non-informative auxiliary domains, which demonstrates the robustness of the proposed method and its ability to detect informative auxiliary subgroups. As the number of informative auxiliary domains card(A) increases, all metrics of the proposed method are greatly improved as expected. We note that the transfer learning method tlgmm completely failed, as it can only handle two-subgroup GGMMs. Therefore, it performs poorly in all settings, even worse than single-domain learning. In challenging Example 2, where the informative auxiliary domains only have the local similarity, the proposed method still significantly outperforms other competitors, even though each target subgroup corresponds to only one informative auxiliary subgroup, which is trapped in a large number of non-informative auxiliary subgroups. It exhibits extremely strong robustness against strong interference and effectiveness in handling the proposed auxiliary situations with the local similarity.

**Table 1:** Averaged metrics and their standard deviation in parenthesis for Example 2.

Network	Methods	Per	*L*	CE	MSE-Mean	MSE-Precision	TPR	FPR
Tridiagonal	Proposed	0.84	3.3900 (0.9309)	0.0071 (0.0183)	0.1233 (0.0943)	1.2414 (0.1031)	0.8426 (0.1560)	0.0351 (0.0097)
	tlgmm	0	2.0000 (0.0000)	0.2226 (0.0002)	2.1577 (0.0514)	4.1801 (0.0798)	–	–
	HeteroGGM	0.84	3.3900 (0.9309)	0.0130 (0.0313)	0.1701 (0.0878)	2.0709 (1.1535)	0.8116 (0.0297)	0.1779 (0.0406)
	SCAN	1	3.0000 (0.0000)	0.0000 (0.0000)	0.5365 (0.0279)	1.2868 (0.0994)	0.8068 (0.1172)	0.0203 (0.0034)
	SCAN.v	0	2.0000 (0.0000)	0.2226 (0.0003)	2.3244 (0.0316)	1.6102 (0.0572)	0.7940 (0.1203)	0.0280 (0.0045)
	JGL	0	2.0000 (0.0000)	0.2226 (0.0000)	2.2844 (0.0229)	1.5106 (0.0374)	0.8104 (0.0579)	0.1038 (0.0319)
Power law	Proposed	0.69	3.8700 (1.3307)	0.0118 (0.0257)	0.1333 (0.0965)	2.6296 (0.1628)	0.9698 (0.0384)	0.0103 (0.0032)
	tlgmm	0	2.0000 (0.0000)	0.2227 (0.0004)	2.1589 (0.0590)	8.0198 (0.1197)	–	–
	HeteroGGM	0.69	3.8700 (1.3307)	0.0404 (0.0624)	0.1771 (0.0886)	3.5765 (0.3082)	0.8658 (0.0398)	0.1461 (0.0443)
	SCAN	1	3.0000 (0.0000)	0.0000 (0.0000)	0.6512 (0.0412)	4.6900 (0.0321)	0.8326 (0.0128)	0.0341 (0.0020)
	SCAN.v	0	2.0000 (0.0000)	0.2226 (0.0000)	2.3806 (0.0333)	6.7911 (0.1226)	0.6464 (0.0263)	0.0335 (0.0017)
	JGL	0	2.0000 (0.0000)	0.2230 (0.0010)	2.2764 (0.0219)	5.7596 (0.1455)	0.8229 (0.0220)	0.1785 (0.0086)

More simulations and corresponding discussions, including settings with imbalanced subgroups, nearest-neighbor network structures, misspecified models, and large discrepancies in subgroup numbers between target and auxiliary domains, have been investigated and are presented in Section E, available as supplementary data at *Bioinformatics* online.

### 3.5 Real data analysis

Cutaneous T-cell lymphoma (CTCL) is cancer originating in white blood cells, primarily thought to originate from mutations in T cells. The two main subtypes are mycosis fungoides (MF) and Sézary syndrome (SS), both derived from mature skin-homing CD4+ T cells. Compared to MF, SS is rarer and characterized by circulating malignant cells with extensive skin involvement, associated with a poor 5-year survival rate. Treatments for advanced SS are often ineffective. Single-cell gene sequencing studies (Buus *et al.* 2018, Borcherding *et al.* 2023) have revealed heterogeneity within SS, suggesting that a deeper understanding of clonal malignant population differences in CTCL could inform more effective treatment strategies.

This study focuses on analyzing the heterogeneity of CD4+ T cells in SS. Borcherding *et al.* (2019) and Borcherding *et al.* (2023) generated four single-cell RNA sequencing datasets of CD4+ T cells in SS: (1) patient nonmalignant CD4+ T cells, (2) patient malignant CD4+ T cells, (3) nonmalignant CD4+ T cells from patients progressed on therapy, and (4) malignant CD4+ T cells from patients progressed on therapy. The respective sample sizes are 4486, 3531, 3725, and 3407. Given their rarity and our primary interest, the malignant CD4+ T cells from therapy-progressed patients serve as our target domain, while the remaining datasets function as auxiliary domains. Additionally, Song *et al.* (2023) reported an unclassified T-cell lymphoma subtype, providing CD4+ T cell data from bone marrow with a sample size of 9223 and peripheral blood with a sample size of 5907; these datasets are also included as auxiliary domains. The analyzed data were sourced directly from CDCP (Li *et al.* 2021), a platform integrating published single-cell datasets, which can be downloaded at https://db.cngb.org/cdcp/dataset/SCDS0000066 (Sézary syndrome data) and https://db.cngb.org/cdcp/dataset/SCDS0000569 (unclassified T-cell lymphoma data). While the original data contained expression levels for over 10 000 genes, many show minimal activity in CTCL. To focus our study, we restricted analysis to genes within the T-cell lymphoma pathway (KEGG, Kanehisa and Goto 2000). Gene expression levels were quantified as ${\;\text{log}\;}_{10}(1+x)$, where *x* represents transcripts-per-million (TPM) values. After excluding genes with missing data or standard deviations below 0.1, analysis proceeded on 61 genes.

**Figure 3:**
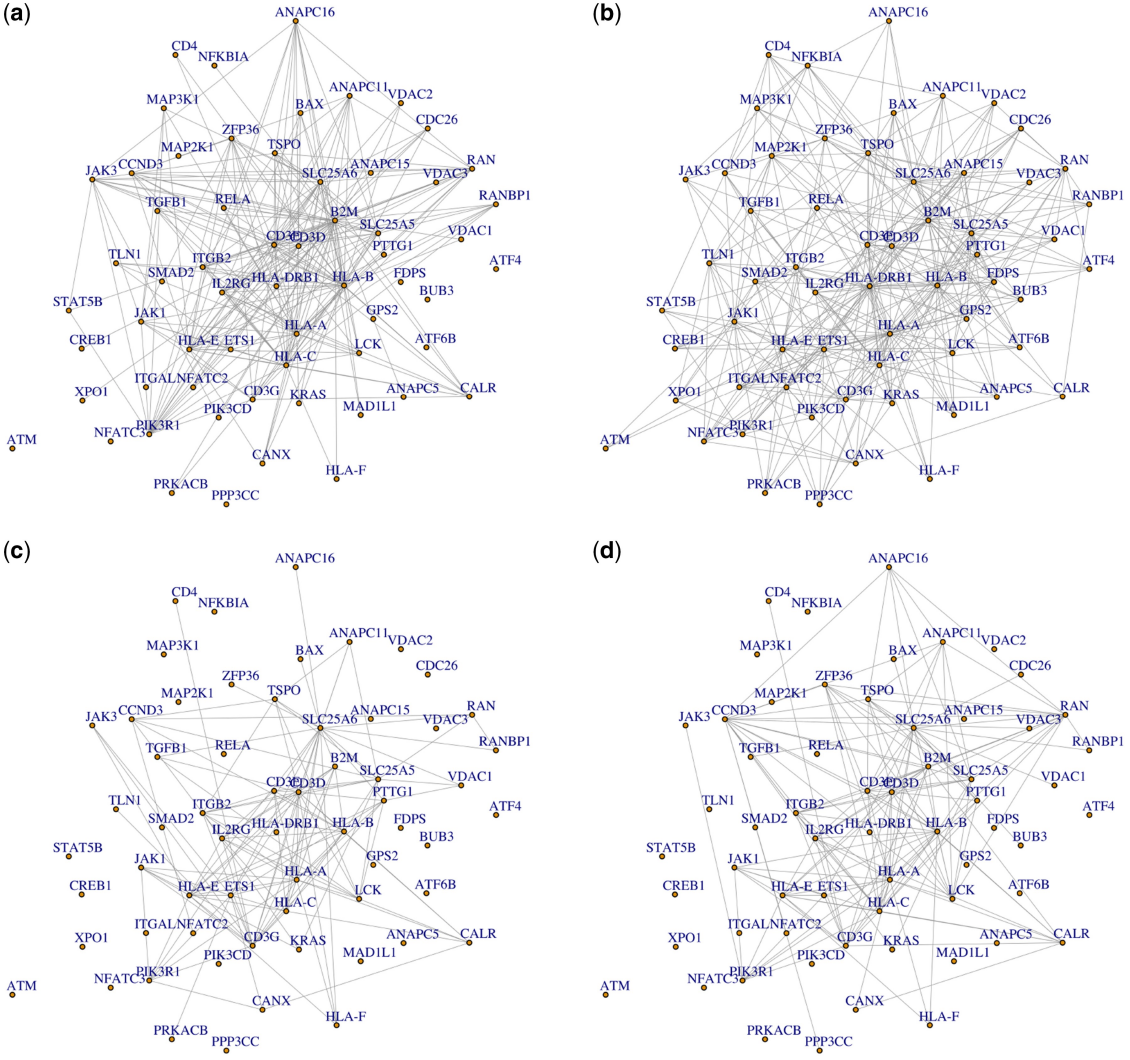
The identified subgrouping network structures. (a and b) The network structures of Subgroup 1 before and after transfer, respectively. (c and d) The network structures of Subgroups 2 and 3, respectively.

Using LtransHeteroGGM, three subgroups are identified, with sizes 379, 1636, and 1392, respectively. Only the learning parameters in Subgroup 1 are transferred, where the informative domain is from the malignant CD4+ T cells without therapy, while there is no informative subgroup of Subgroups 2 and 3, confirming the strong heterogeneity of real single-cell datasets. Figure 3 shows the network structures of Subgroup 1 before and after transfer, and the network structures of Subgroups 2 and 3. It is easy to observe that the three identified network structures, with 382, 127, and 154 edges, respectively, are significantly different. The hub genes identified in the three subgroups also exhibit slight variations; e.g. HLA-DRB1 and JAK1 are uniquely identified as hub genes in Subgroup 1, and CCND3 is uniquely identified as a hub gene in Subgroup 3. Addition, a table comparing network characteristics in Subgroup 1 before and after transfer is summarized in Table S14, available as supplementary data at *Bioinformatics* online, and a heatmap of edges in Subgroup 1 before and after transfer is presented in Fig. S4, available as supplementary data at *Bioinformatics* online.

For the results in Subgroup 1, a subgroup benefiting from transfer learning, we first consider the commonalities before and after using LtransHeteroGGM. We focus on eight hub genes with node degree both ranked among the top 10 before and after information transfer, B2M, HLA-A, HLA-B, HLA-C, CD3D, CD3E, SLC25A5, and SLC25A6. It very interesting to note that these eight genes can be classified into three categories, where the proteins encoded by B2M, HLA-A, HLA-B, and HLA-C are components of MHC class I molecules, the proteins encoded by CD3D and CD3E are main components of T-cell receptor-CD3 complex, and the proteins encoded by SLC25A5 and SLC25A6 are main components of ADP/ATP translocase (ANT). Some existing works have shown the biological significance of these genes, with details in Section D.2, available as supplementary data at *Bioinformatics* online. Notably, certain genes exhibit contrasting topological roles, functioning as hub nodes after information transfer, while transitioning to peripheral nodes before information transfer, such as HLA-DRB1 and ETS1, which demonstrate critical regulatory roles in Sézary syndrome pathogenesis. A detailed description of their biological significance can be found in the Supporting Information, available as supplementary data at *Bioinformatics* online.

In addition to network structures, we further attempt to identify the cell types corresponding to these three subgroups. Some marker genes of SS have been widely reported in literature, including CCR4, DUSP1, GPR15, ICAM2, JUNB, KIR3DL2, PLS3, ITGB1, GATA3, NEDD4L, LAT, MGAT4A, PDCD1, SKAP1, and TOX (Borcherding *et al.* 2019), they should be highly expressed in SS cells. Their average expression levels in each subgroup are summarized in Fig. S2b, available as supplementary data at *Bioinformatics* online. It is noted that these genes are not in T-cell lymphoma pathway; as such, there is no overfitting problem. It is very interesting to note that the average expression levels of all marker genes in Subgroup 1 are significantly smaller than those in Subgroup 2, and the expression levels in Subgroup 3 were lying between Subgroup 1 and Subgroup 2 in magnitude, therefore, we speculate that cells in Subgroup 1, Subgroup 3, and Subgroup 2 may embody three consecutive stages of SS development. Also, it has been verified that gene–gene network complexity is reduced in the transition from normal to cancer (Arshad and McDonald 2021), which is consistent with our speculated conclusion. These marker genes with significant expression differences also exert indispensable regulatory effects on SS pathogenesis. For example, CCR4 plays a crucial role in the process of T cell extravasation to the skin (Ferenczi *et al.* 2002). TOX is aberrantly expressed in primary Sézary cells, correlating with the risk of disease-specific mortality, and its knockdown promotes apoptosis and reduces cell proliferation in CTCL cells (Huang *et al.* 2015).

Moreover, we present the solution paths (Ma and Huang 2017) of the number of subgroups and the F-norm of learned parameters against λ, where λ is the parameter controlling the number of subgroups, the solution paths are plotted in Figs. S5 and S6, available as supplementary data at *Bioinformatics* online. It is shown that as λ increases, nearby data subgroups merge gradually, eventually forming a single group. We also discuss how the performance of the proposed method will be affected if the number of subgroups is fixed. The number of subgroups is set to 2, 4, and 5, respectively, and the conclusions are summarized in Section F.3, available as supplementary data at *Bioinformatics* online.

Finally, we evaluate the stability of clustering of some competitors with details in the last paragraph of Section D.2, available as supplementary data at *Bioinformatics* online, and the proposed method attains satisfactory stability performance.

## 4 Discussion

This article proposes LtransHeteroGGM, which integrates local similarities between the target domain and auxiliary domains to improve estimation accuracy and allows different subgroup numbers and partially similar parameters between domains. The simulation and real data analysis have demonstrated satisfactory computational properties.

Exploring the theoretical properties of the proposed method is a promising direction. The profound difficulties stem directly from fundamental and unresolved gaps in the core theory for single-dataset high-dimensional GGMM, including estimation consistency under the max norm and clustering consistency. Crucially, the development of statistical inference theory for such high-dimensional models is itself a major unsolved problem within the field (Hao *et al.* 2018), so that extending these critical theoretical foundations to the more complex transfer learning setting poses an exceptionally difficult, multi-layered challenge. Resolving these deep theoretical gaps, therefore, represents a highly valuable and promising direction for substantial future research.

An additional valuable perspective on transfer learning methodologies involves utilizing subgroup information inferred from the auxiliary domain as prior knowledge (Mitra *et al.* 2013, Zhang *et al.* 2016). This prior information may contain both informative and uninformative (potentially even detrimental) components. By employing a fused lasso penalty (Zhang *et al.* 2009, 2011, Fu *et al.* 2024) to penalize differences between these priors and the target parameters, one can effectively identify and select informative prior knowledge, thereby facilitating transfer learning. This approach represents a highly promising research avenue. Indeed, the core concept of penalizing discrepancies within existing transfer learning frameworks (Li *et al.* 2022a) also embodies this same underlying principle.

In addition, the proposed transfer learning ideas and model selection have some subtle connections. Model selection is the process of choosing the best model from a set of candidates for a specific task, focusing on finding the optimal balance between model fitness and its complexity to achieve better generalization performance. The regularization model selection method, represented by lasso or fused lasso (Friedman *et al.* 2008, Zhang and Wang 2012, Tian *et al.* 2014), shares a profound conceptual connection with the transfer learning approach we propose. Fundamentally, these penalized regularization techniques achieve model selection by shrinking weak coefficients to zero. In parallel, our proposed transfer learning framework, encompassing established methods, operates essentially by penalizing the discrepancy between parameters in the auxiliary and target domains. This penalty effectively drives minor domain differences toward zero, enabling the crucial selection of informative auxiliary domains, thus, both methodologies embody the core philosophy of penalization. Traditional regularization penalizes and shrinks the model parameters themselves; however, transfer learning penalizes and shrinks the differences between parameters across domains.

## Supplementary Material

btag057_Supplementary_Data
