# Supplementary material for: LtransHeteroGGM: local transfer learning for Gaussian graphical model-based heterogeneity analysis
Source: Bioinformatics. 2026 Feb 4;42(2):btag057. doi: 10.1093/bioinformatics/btag057 (PMC12944826; doi:10.1093/bioinformatics/btag057)
Supplement: btag057_Supplementary_Data [file btag057_supplementary_data.pdf]

Supporting Information for "LtransHeteroGGM: Local transfer learning for Gaussian graphical model-based heterogeneity analysis"

Chengye Li, Hongwei Ma, and Mingyang Ren

A Preliminaries

Denote $\|\boldsymbol{u}\|_q$ as the l_q-norm of a vector $\boldsymbol{u}$, for $q \geqslant 0$. For a matrix $\boldsymbol{A} = (A_{ij})_{1\leqslant i,j\leqslant p}$, let $\|\boldsymbol{A}\|_{q,\infty} = \max_{1\leqslant j\leqslant p} \|\boldsymbol{A}_j\|_q$, $\|\boldsymbol{A}\|_1 = \sum_{j=1}^{p} \|\boldsymbol{A}_j\|_1$, $\|\boldsymbol{A}\|_{1,\text{off}} = \sum_{1\leqslant i\neq j\leqslant p} |A_{ij}|$, and $\|\boldsymbol{A}\|_F$ be the Frobenius norm of $\boldsymbol{A}$. Before introducing the proposed LtransHeteroGGM, this section introduces Gaussian graphical mixture models (GGMM) for the single domain, given their fundamental role in this article.

Gaussian graphical mixture models. Assume that the available independent samples $\boldsymbol{x}$ follows a Gaussian mixture distribution $\sum_{l=1}^{L} \pi_l^* f_l\left(\boldsymbol{x}; \boldsymbol{\mu}_l^*, (\boldsymbol{\Theta}_l^*)^{-1}\right)$, where

$$f_l\left(\boldsymbol{x}; \boldsymbol{\mu}_l^*, (\boldsymbol{\Theta}_l^*)^{-1}\right) = (2\pi)^{-p/2} \left|\boldsymbol{\Theta}_l^*\right|^{1/2} \exp\left\{-\frac{1}{2}\left(\boldsymbol{x} - \boldsymbol{\mu}_l^*\right)^\top \boldsymbol{\Theta}_l^* \left(\boldsymbol{x} - \boldsymbol{\mu}_l^*\right)\right\} \quad \text{(S.1)}$$

is the Gaussian density of the l-th subgroup with unknown mean vector $\boldsymbol{\mu}_l^*$ and precision matrix $\boldsymbol{\Theta}_l^*$, L is the number of subgroups, π_l^*'s are unknown mixture probabilities, and the subgrouping memberships are also unknown. To estimate $\{\boldsymbol{\mu}_l^*, \boldsymbol{\Theta}_l^*\}_{l=1}^{L}$ of interest, a unified objective function is well established under the known L, $\frac{1}{n}\sum_{i=1}^{n} \log\left(\sum_{l=1}^{L} \pi_l f_l\left(\boldsymbol{x}_i; \boldsymbol{\mu}_l, \boldsymbol{\Theta}_l^{-1}\right)\right) - \mathcal{P}_{\mu,\Theta}$, where $\mathcal{P}_{\mu,\Theta} = \sum_{l=1}^{L}\sum_{j=1}^{p} p(|\mu_{lj}|, \lambda_1') + \sum_{l=1}^{L}\sum_{i\neq j} p(|\theta_{lij}|, \lambda_2') + \sum_{i\neq j} p((\sum_{l=1}^{L}\theta_{lij}^2)^{1/2}, \lambda_3') + \sum_{l<l'}\sum_{1\leqslant i,j\leqslant p} p(|\theta_{lij} - \theta_{l'ij}|, \lambda_4')$, and $p(\cdot, \lambda')$ can be any sparsity-induced penalty function. Early GGMMs (Pan and Shen, 2007) focused on the variable selection of mean vectors with

$\lambda_2' = \lambda_3' = \lambda_4' = 0$, requiring all precision matrices to be diagonal. This assumption has been relaxed in Zhou et al. (2009) with $\lambda_3' = \lambda_4' = 0$, Gao et al. (2016) with $\lambda_3' = 0$, and Hao et al. (2018) with $\lambda_4' = 0$.

To address more challenging cases with an unknown number of subgroups, Ren et al. (2022) proposed HeteroGGM approach by introducing the fusion penalty technique. Specifically, although it is hard to know L a priori, their "upper bound" M can be easily specified, either based on some professional knowledge or the maximum number of subgroups desirable. To be cautious, M can be set as a relatively large number. Denote $\boldsymbol{\Omega}_m = \operatorname{vec}(\boldsymbol{\mu}_m, \boldsymbol{\Theta}_m) = (\mu_{m1}, \ldots, \mu_{mp}, \theta_{m11}, \ldots, \theta_{mp1}, \ldots, \theta_{m1p}, \ldots, \theta_{mpp}) \in \mathbb{R}^{p^2+p}$, and then $\{\widehat{\boldsymbol{\Omega}}_m, \widehat{\pi}_m\}_{m=1}^M$ can be estimated by $\operatorname{argmax}_{\{\boldsymbol{\Omega}_m, \pi_m\}_{m=1}^M} \frac{1}{n}\sum_{i=1}^n \log\left(\sum_{m=1}^M \pi_m f_m\left(\boldsymbol{x}_i; \boldsymbol{\mu}_m, \boldsymbol{\Theta}_m^{-1}\right)\right) - \sum_{m=1}^M \sum_{j=1}^p p(|\mu_{mj}|, \lambda_1') - \sum_{m=1}^M \sum_{i\neq j} p(|\theta_{mij}|, \lambda_2') - \sum_{m<m'} p\left(\left(\|\boldsymbol{\mu}_m - \boldsymbol{\mu}_{m'}\|_2^2 + \|\boldsymbol{\Theta}_m - \boldsymbol{\Theta}_{m'}\|_F^2\right)^{1/2}, \lambda_3'\right)$. The third penalty shrinks differences among the M subgroups and encourages equality, that is, a smaller number of subgroups. Denote $\{\widehat{\boldsymbol{\Upsilon}}_l\}_{l=1}^{\widehat{L}}$ as the distinct values of $\{\widehat{\boldsymbol{\Omega}}_m\}_{m=1}^M$, that is, $\{m : \widehat{\boldsymbol{\Omega}}_m \equiv \widehat{\boldsymbol{\Upsilon}}_l, m = 1, \cdots, M\}_{l=1,\cdots,\widehat{L}}$ constitutes a partition of $\{1, \cdots, M\}$. Then it is estimated that there are $\widehat{L}$ subgroups with estimated means, precision matrices, and mixture probabilities extracted in $\{\widehat{\boldsymbol{\Upsilon}}_l\}_{l=1}^{\widehat{L}}$. The HeteroGGM approach involves three tuning parameters $(\lambda_1', \lambda_2', \lambda_3')$. The tuning parameter selection is conducted via optimizing the adaptive BIC-type criterion: $-2\sum_{i=1}^n \log\left\{\sum_{l=1}^{\widehat{L}} \widehat{\pi}_l f_l\left(\boldsymbol{x}_i; \widehat{\boldsymbol{\mu}}_l, (\widehat{\boldsymbol{\Theta}}_l)^{-1}\right)\right\} + \sum_{l=1}^{\widehat{L}} (\log n \cdot s_{1l} + 2 s_{2l})$, where $s_{1l} = |\{j : \widehat{\mu}_{lj} \neq 0, 1 \leqslant j \leqslant p\}|$, $s_{2l} = |\{(i,j) : \widehat{\theta}_{lij} \neq 0, 1 \leqslant i < j \leqslant p\}|$, for $l = 1, \cdots, \widehat{K}_0$. This method is an important foundation for the initialization of the proposed LtransHeteroGGM.

B More information about the proposed algorithm

B.1 Algorithm for Step 3

Recall that there are two optimization problems in Step 3,

$$\widehat{\boldsymbol{\Delta}}_l = \underset{\boldsymbol{\Delta}}{\operatorname{argmin}} \ \Big(\frac{1}{2}\operatorname{tr}\{\boldsymbol{\Delta}^\top\boldsymbol{\Delta}\} - \operatorname{tr}\{(\widehat{\boldsymbol{\Theta}}_l^{(0)}\widehat{\boldsymbol{\Sigma}}_l^{\mathcal{A}} - \boldsymbol{I}_p)^\top\boldsymbol{\Delta}\} + \lambda_1\|\boldsymbol{\Delta}\|_1\Big), \tag{S.2}$$

$$\widehat{\boldsymbol{\Theta}}_l = \underset{\boldsymbol{\Theta}_l}{\operatorname{argmin}} \ \Big(\frac{1}{2}\operatorname{tr}\{\boldsymbol{\Theta}_l^\top\widehat{\boldsymbol{\Sigma}}_l^{\mathcal{A}}\boldsymbol{\Theta}_l\} - \operatorname{tr}\{(\widehat{\boldsymbol{\Delta}}_l^\top + \boldsymbol{I}_p)\boldsymbol{\Theta}_l\} + \lambda_2\|\boldsymbol{\Theta}_l\|_{1,\text{off}}\Big), \tag{S.3}$$

for $l = 1, \cdots, \widehat{L}$. Note that the algorithms for each subgroup l are completely consistent. For simplicity, we will ignore the subscript l in the following algorithm description.

The optimization tasks in both (S.2) and (S.3) are convex and can be efficiently solved via coordinate descent. Specifically, for estimating $\widehat{\boldsymbol{\Delta}}$, (S.2) can be rewritten as

$$\frac{1}{2}\sum_{1\leqslant i,j\leqslant p}\Delta_{ij}^2 - \sum_{1\leqslant i,j\leqslant p}\widehat{\Delta}_{ij}^{(\check{k})}\Delta_{ij} + \lambda_1\sum_{1\leqslant i,j\leqslant p}|\Delta_{ij}|,$$

where Δ_{ij} and $\widehat{\Delta}_{ij}^{(\check{k})}$ are the (i,j) entries of $\boldsymbol{\Delta}$ and $\widehat{\boldsymbol{\Delta}}^{(\check{k})}$, respectively. It can be separated into p^2 lasso-type optimizations; that is, for any i and j,

$$\widehat{\Delta}_{ij} = \underset{\Delta}{\operatorname{argmin}}\left\{\frac{1}{2}(\Delta - \widehat{\Delta}_{ij}^{(\check{k})})^2 + \lambda_1|\Delta|\right\} = \mathcal{T}(\widehat{\Delta}_{ij}^{(\check{k})}, \lambda_1), \tag{S.4}$$

where $\mathcal{T}(z,\lambda) = \operatorname{sign}(z)\max(0, |z| - \lambda)$.

For estimating $\widehat{\boldsymbol{\Theta}}$, (S.3) can be rewritten as

$$\sum_{1\leqslant j\leqslant p}\left\{\frac{1}{2}\boldsymbol{\Theta}_j^\top\widehat{\boldsymbol{\Sigma}}^{(k)}\boldsymbol{\Theta}_j - \boldsymbol{\Theta}_j^\top(\widehat{\boldsymbol{\Delta}}_j + [\boldsymbol{I}_p]_j) + \lambda_2\|\boldsymbol{\Theta}_j\|_1 - \lambda_2|\boldsymbol{\Theta}_{jj}|\right\},$$

where $\boldsymbol{\Theta}_j$ and $[\boldsymbol{I}_p]_j$ are the j-th columns of $\boldsymbol{\Theta}$ and $\boldsymbol{I}_p$, respectively. It can be separated into

p optimizations; that is, for any j,

$$\widehat{\mathbf{\Theta}}_j = \operatorname*{argmin}_{\boldsymbol{\theta}} \left\{ \frac{1}{2} \boldsymbol{\theta}^\top \widehat{\mathbf{\Sigma}}^{(k)} \boldsymbol{\theta} - \boldsymbol{\theta}^\top (\widehat{\mathbf{\Delta}}_j + [\boldsymbol{I}_p]_j) + \lambda_2 \|\boldsymbol{\theta}_{-j}\|_1 \right\}, \tag{S.5}$$

where $\boldsymbol{\theta}_{-j}$ is the sub-vector of $\boldsymbol{\theta}$ with the j-th component removed.

For the optimization of (S.5), we adopt the coordinate descent algorithm. Particularly, at iteration $t+1$, the updating formula of θ_i, i-th component of $\boldsymbol{\theta}$, with other components $\{\theta_{i'}^{(t+1)}, i' < i;\ \theta_{i'}^{(t)}, i' > i\}$ fixed, are

$$\theta_i^{(t+1)} = [\widehat{\mathbf{\Sigma}}_{ii}^{(k)}]^{-1} \mathcal{T}(\iota^{(t)}, \lambda_2 I(i \neq j)), \text{ for } i = 1, \cdots, p,$$

where $\iota^{(t)} = [\widehat{\mathbf{\Delta}} + \boldsymbol{I}_p]_{ij} - \sum_{i'<i} \theta_{i'}^{(t+1)} \widehat{\mathbf{\Sigma}}_{ii'}^{(k)} - \sum_{i'>i} \theta_{i'}^{(t)} \widehat{\mathbf{\Sigma}}_{ii'}^{(k)}$.

As computational remarks, the explicit solution derived in each step makes the algorithm very efficient. The initial values of $\boldsymbol{\theta}$ are set as $\widehat{\mathbf{\Theta}}_j^{(0)}$. Note that these developments are specifically for the Lasso penalty, and optimization with other penalties may require minor modifications. Convergence properties of the algorithm can be guaranteed, thanks to the convexity of the objective function. As for the tuning parameter selection, we set $\lambda_1 = 2\|\widehat{\mathbf{\Theta}}^{(0)}\|_{1,\infty}\sqrt{\frac{\log p}{n}}$, following Li et al. (2022b). For λ_2, it is suggested to be determined via minimizing a BIC-type criterion, $\frac{1}{2}\operatorname{tr}\{\widehat{\mathbf{\Theta}}^\top \widehat{\mathbf{\Sigma}}^{(k)} \widehat{\mathbf{\Theta}}\} - \operatorname{tr}\{(\widehat{\mathbf{\Delta}}^\top + \boldsymbol{I}_p)\widehat{\mathbf{\Theta}}\} + \frac{\log n}{n}\|\widehat{\mathbf{\Theta}}\|_0$.

Table S1: A summary table comparing HeteroGGM and LtransHeteroGGM

	HeteroGGM	LtransHeteroGGM
Mixture Gaussian distribution structure	✓	✓
EM framework	✓	✓
Heterogeneity within a single dataset	✓	✓
Initialization	K-means	HeteroGGM
Multi-source data	×	✓
Heterogeneity between datasets	×	✓
Local similarity	×	✓
Adaptive integration for auxiliary domains	×	✓
Subgroup-level local transfer	×	✓

B.2 Rationale of adaptive weights in Step 2

Recall that for each target subgroup l, its roughly estimated informative auxiliary set is $\widehat{\mathcal{A}}_l^{\Theta}$, then the auxiliary covariance matrix is aggregated through adaptive weighting, $\widehat{\mathbf{\Sigma}}_l^{\mathcal{A}} = \sum_{(l',k)\in\widehat{\mathcal{A}}_l^{\Theta}} \alpha_{kl'} \widetilde{\mathbf{\Sigma}}_{l'}^{(k)}$, with $\alpha_{kl'} = \frac{n_{kl'}/\|\widehat{\mathbf{\Delta}}_{ll'}^{(k)}\|_{1,\infty}}{\sum_{(l',k)\in\widehat{\mathcal{A}}_l^{\Theta}}(n_{kl'}/\|\widehat{\mathbf{\Delta}}_{ll'}^{(k)}\|_{1,\infty})}$.

To provide the intuitive insights, we can begin with a natural choice of the weights, which is widely used in existing transfer learning literature (Li et al., 2022b; He et al., 2022),

$$\alpha_{kl'} = \frac{n_{kl'}}{\sum_{(l',k)\in\widehat{\mathcal{A}}_l^{\Theta}} n_{kl'}}.$$

This approach assigns weights proportional to auxiliary domain sample sizes, based on the rationale that domains with larger samples should contribute more significantly. However, this method fails to account for inter-domain similarities. A critical limitation arises when large, non-informative auxiliary subgroups exhibit marked dissimilarity from the target domain. In such scenarios, it may simultaneously counteract or even dominate potential improvements achievable through informative, positively correlated auxiliary subgroups. For example, if non-informative auxiliary subgroups exist, adopting this widely used weighting scheme in existing transfer learning literature, which relies solely on sample size while disregarding the degree of inter-domain similarity, will construct an aggregated auxiliary covariance matrix that deviates from the target subgroup. Consequently, during subsequent parameter transfer, the estimates obtained via transfer learning methods will exhibit significant deviation from the true values of the target subgroup. This will result in performance inferior to that achieved using the target subgroup data alone—a phenomenon known as negative transfer.

To address this challenge, we propose data-adaptive weighting for auxiliary covariance matrices. These weights simultaneously incorporate auxiliary subgroup sample sizes and

estimated divergences from the target subgroup. Particularly, we set

$$\alpha_{kl'} = \frac{n_{kl'}/\|\widehat{\boldsymbol{\Delta}}_{ll'}^{(k)}\|_{1,\infty}}{\sum_{(l',k)\in\widehat{\mathcal{A}}_l^{\Theta}}(n_{kl'}/\|\widehat{\boldsymbol{\Delta}}_{ll'}^{(k)}\|_{1,\infty})},$$

where $\widehat{\boldsymbol{\Delta}}_{ll'}^{(k)} = \widehat{\boldsymbol{\Theta}}_l^{(0)}\widetilde{\boldsymbol{\Sigma}}_{l'}^{(k)} - \boldsymbol{I}_p$ is the divergence matrix between the l-th target subgroup and the l'-th subgroup in the k-th auxiliary domains. This design ensures that among auxiliary subgroups of comparable size, greater weight is assigned to those exhibiting higher similarity to the target subgroup. Conversely, weights for subgroups with extreme dissimilarity can asymptotically approach zero (under the condition of sufficient separability between the non-informative auxiliary subgroup and the target subgroup, this can be theoretically guaranteed according to the proofs of Theorem 4 in Ren et al. (2024)), thereby mitigating negative transfer through adaptive penalization. We note that the choice of norm for quantifying domain difference is not critical; the specified L_1-norm primarily maintains consistency with the local transfer step and can be substituted with other norms via minor modifications. It is also interesting to note that even with these adaptive weights, a pre-screening step remains essential to safeguard against scenarios where all auxiliary subgroups are non-informative.

B.3 More discussions on the hard-threshold cutoff and the rate of h_Θ

Note that for subgroup l of the target domain, the informative auxiliary set for precision matrix is defined as $\mathcal{A}_l^{\Theta} = \{(l',k) : \|\boldsymbol{\Delta}_{ll'}^{(k)*}\|_{1,\infty} + \|(\boldsymbol{\Delta}_{ll'}^{(k)*})^{\top}\|_{1,\infty} \leqslant h_\Theta\}$ for some small $h_\Theta > 0$, where $\boldsymbol{\Delta}_{ll'}^{(k)*} = \boldsymbol{\Theta}_l^*\boldsymbol{\Sigma}_{l'}^{(k)*} - \boldsymbol{I}_p$ measure the difference between the l-th target subgroup and the l'-th auxiliary subgroup in the k-th domain, respectively. Within the well-established theoretical framework for transfer learning in single Gaussian graphical models (Li et al., 2022b), this parameter h_Θ quantifies the transferable information from the auxiliary do-

mains, and $h_\Theta = O(s\sqrt{\frac{\log p}{n_{0l}}})$ with $s = \|\boldsymbol{\Theta}_l^{(0)}\|_{0,\infty}$. When we set the cutoff threshold, this rate is referenced, sharing the same spirit as Tian et al. (2022); Tian and Feng (2022). Therefore, provided the distance between non-informative auxiliary subgroups and the target subgroup is significantly larger than this relatively small rate (a condition typically satisfied and referred to as the *detectable condition*), the chosen cutoff threshold generally remains effective and robust. Crucially, this robustness holds irrespective of both the number of auxiliary subgroups and the complexity of their structural configurations.

Moreover, subsequent to the initial truncation-based pre-screening, we augment the procedure with an adaptive weighting scheme for aggregating the auxiliary subgroups. This step is designed to further mitigate the influence of parameters from non-informative auxiliary sources. This dual-safeguard mechanism has been theoretically validated to be effective (Ren et al., 2024).

B.4 Local transfer for the estimation of the mean

The main concern in Gaussian graphical models is the precision matrix; nevertheless, a similar procedure can be constructed if we also want to improve the estimation of the mean. Specifically, in the pre-screening and adaptive weighting step, for l-th target subgroup, reserve the auxiliary subgroups such that $(l', k) \in \widehat{\mathcal{A}}_l^{\mu} = \{(l', k) : \|\widehat{\boldsymbol{\delta}}_{ll'}^{(k)}\|_1 \leqslant c\|\widehat{\boldsymbol{\mu}}_l^{(0)}\|_0\sqrt{\frac{\log p}{n_{0l}}}\}$ with $\widehat{\boldsymbol{\delta}}_{ll'}^{(k)} = \widehat{\boldsymbol{\mu}}_l^{(0)} - \widehat{\boldsymbol{\mu}}_{l'}^{(k)}$. The weighted mean vector of the retained auxiliary subgroups, $\widehat{\boldsymbol{\mu}}_l^{\mathcal{A}}$, can be obtained by a similar adaptive weighting. In the local transfer step, we can first conduct the adaptive difference parameters of mean vectors,

$$\widehat{\boldsymbol{\delta}}_l = \underset{\boldsymbol{\delta}}{\operatorname{argmin}} \frac{1}{2}\frac{\sum_{i=1}^n \widehat{\gamma}_{il}\|\boldsymbol{x}_i - \widehat{\boldsymbol{\mu}}_l^{\mathcal{A}} - \boldsymbol{\delta}\|_2^2}{\sum_{i=1}^n \widehat{\gamma}_{il}} + \widetilde{\lambda}_l\|\boldsymbol{\delta}\|_1,$$

and then the mean estimator can be obtained as $\widehat{\boldsymbol{\mu}}_l = \widehat{\boldsymbol{\mu}}_l^{\mathcal{A}} + \widehat{\boldsymbol{\delta}}_l$.

C Details of EM algorithm for Initialization

For the initialization step, it is necessary to apply the penalized EM algorithm to all domains separately to obtain initial parameter estimators. In this section, we introduce the E-step and M-step of the penalized EM algorithm as well as the stopping criteria, summarized in Algorithm S1. Note that the penalized EM algorithm is applicable to all target and auxiliary domains, so superscripts (k) for all parameters referring to domain numbers are ignored in this section.

Recall that the objective function is:

$$\mathcal{L}(\boldsymbol{\Omega}, \boldsymbol{\pi}|\boldsymbol{X}) := \frac{1}{n}\sum_{i=1}^{n} \log\left(\sum_{k=1}^{K} \pi_k f_k\left(\boldsymbol{x}_i; \boldsymbol{\mu}_k, \boldsymbol{\Theta}_k^{-1}\right)\right) - \mathcal{P}(\boldsymbol{\Omega}), \tag{S.6}$$

where $\boldsymbol{\pi} = (\pi_1, \cdots, \pi_K)^\top$, $\boldsymbol{\Theta}_k = \boldsymbol{\Sigma}_k^{-1}$ is the k-th precision matrix with the ij-th entry θ_{kij}, $\boldsymbol{\Omega} = (\boldsymbol{\Omega}_1^\top, \cdots, \boldsymbol{\Omega}_K^\top)^\top$, $\boldsymbol{\Omega}_k = \mathrm{vec}\left(\boldsymbol{\mu}_k, \boldsymbol{\Theta}_k\right) = (\mu_{k1}, \ldots, \mu_{kp}, \theta_{k11}, \ldots, \theta_{kp1}, \ldots, \theta_{k1p}, \ldots, \theta_{kpp}) \in \mathbb{R}^{p^2+p}$,

$$\begin{aligned} \mathcal{P}(\boldsymbol{\Omega}) = &\sum_{k=1}^{K}\sum_{j=1}^{p} p(|\mu_{kj}|, \lambda_1') + \sum_{k=1}^{K}\sum_{i\neq j} p(|\theta_{kij}|, \lambda_2') \\ &+ \sum_{k<k'} p\left(\left(\|\boldsymbol{\mu}_k - \boldsymbol{\mu}_{k'}\|_2^2 + \|\boldsymbol{\Theta}_k - \boldsymbol{\Theta}_{k'}\|_F^2\right)^{1/2}, \lambda_3'\right), \end{aligned} \tag{S.7}$$

and $p(\cdot, \lambda')$ is the MCP function with a tuning parameter $\lambda' > 0$.

In the t-th expectation step of the EM algorithm, the following function needs to be maximized:

$$E_{\boldsymbol{\gamma}|\boldsymbol{X}, \boldsymbol{\Omega}^{(t-1)}}[\mathcal{L}(\boldsymbol{\Omega}|\boldsymbol{X}, \boldsymbol{\gamma})] = \frac{1}{n}\sum_{i=1}^{n}\sum_{k=1}^{K} \gamma_{ik}^{(t)}\left[\log \pi_k + \log f_k\left(\boldsymbol{x}_i; \boldsymbol{\mu}_k, \boldsymbol{\Theta}_k^{-1}\right)\right] - \mathcal{P}(\boldsymbol{\Omega}), \tag{S.8}$$

where $\mathcal{P}(\boldsymbol{\Omega})$ is defined in (S.7), and $\gamma_{ik}^{(t)}$ can be computed based on the parameters $\pi_k^{(t-1)}$,

$\boldsymbol{\mu}_k^{(t-1)}$, and $\boldsymbol{\Theta}_k^{(t-1)}$ obtained in the previous iteration. More specifically,

$$\gamma_{ik}^{(t)} = \frac{\pi_k^{(t-1)} f_k\left(\boldsymbol{x}_i; \boldsymbol{\mu}_k^{(t-1)}, \left(\boldsymbol{\Theta}_k^{(t-1)}\right)^{-1}\right)}{\sum_{k=1}^K \pi_k^{(t-1)} f_k\left(\boldsymbol{x}_i; \boldsymbol{\mu}_k^{(t-1)}, \left(\boldsymbol{\Theta}_k^{(t-1)}\right)^{-1}\right)}. \tag{S.9}$$

In the t-th maximization step, maximizing (S.8) with respect to $\pi_k, \boldsymbol{\mu}_k, \boldsymbol{\Theta}_k$ yields the update of parameters. More specifically, the update of π_k is given by

$$\pi_k^{(t)} = \frac{1}{n}\sum_{i=1}^n \gamma_{ik}^{(t)}. \tag{S.10}$$

For $\boldsymbol{\mu}_k$, it is noted that maximizing (S.8) with respect to $\{\boldsymbol{\mu}\} = \boldsymbol{\mu}_1, \ldots, \boldsymbol{\mu}_K$ is equivalent to solving:

$$\{\boldsymbol{\mu}^{(t)}\} = \underset{\{\boldsymbol{\mu}\}}{\operatorname{argmin}} \left(\frac{1}{2n} \sum_{i=1}^n \sum_{k=1}^K \gamma_{ik}^{(t)} \left\{ (\boldsymbol{x}_i - \boldsymbol{\mu}_k)^\top \boldsymbol{\Theta}_k^{(t-1)} (\boldsymbol{x}_i - \boldsymbol{\mu}_k) \right\} + \mathcal{P}(\boldsymbol{\Omega}) \right). \tag{S.11}$$

For this problem, the local quadratic approximation can be adopted, which can lead to an explicit solution at each iteration. Details can be referred to Ren et al. (2022).

Maximizing (S.8) with respect to $\{\boldsymbol{\Theta}\}$ is equivalent to solving:

$$\{\boldsymbol{\Theta}_k^{(t)}, k = 1, \ldots, K\} = \underset{\{\boldsymbol{\Theta}\}}{\operatorname{argmax}} \left(\sum_{k=1}^K n_k \left[\log\{\det(\boldsymbol{\Theta}_k)\} - \operatorname{tr}\left(\widetilde{\boldsymbol{S}}_k \boldsymbol{\Theta}_k \right) \right] - \mathcal{P}(\{\boldsymbol{\Theta}\}) \right), \tag{S.12}$$

where $n_k = \sum_{i=1}^n \gamma_{ik}^{(t)}$, $\widetilde{\boldsymbol{S}}_k$ is the pseudo sample covariance matrix defined by:

$$\widetilde{\boldsymbol{S}}_k = \frac{\sum_{i=1}^n \gamma_{ik}^{(t)} \left(\boldsymbol{x}_i - \boldsymbol{\mu}_k^{(t)}\right)\left(\boldsymbol{x}_i - \boldsymbol{\mu}_k^{(t)}\right)^\top}{\sum_{i=1}^n \gamma_{ik}^{(t)}},$$

and $\mathcal{P}(\{\boldsymbol{\Theta}\}) = \sum_{k=1}^K \sum_{i \neq j} p(|\theta_{kij}|, \lambda_2') + \sum_{k<k'} p\left((\|\boldsymbol{\mu}_k^{(t)} - \boldsymbol{\mu}_{k'}^{(t)}\|_2^2 + \|\boldsymbol{\Theta}_k - \boldsymbol{\Theta}_{k'}\|_F^2)^{1/2}, \lambda_3' \right)$.

The solution for (S.12) can be effectively obtained using the ADMM technique. More details are provided in Section C.1. Overall, we propose the algorithm summarized in Algorithm S1.

Algorithm S1 for maximizing (S.6)

Input: $\boldsymbol{x}_i, i = 1, \cdots, n$, tuning parameters $\lambda_1', \lambda_2', \lambda_3'$, and K.
Output: The estimated mean vectors and precision matrices.
Initialization: Mean vectors $\boldsymbol{\mu}_k^{(0)}$, positive-definite precision matrices $\boldsymbol{\Theta}_k^{(0)}$, and $\pi_k^{(0)}$ obtained using the K-means method, for $k = 1, \cdots, K$.
Repeat for $t = 1, 2, 3, \ldots$ **as follows**:

1. E-step: Update the subpopulation assignment $\gamma_{ik}^{(t)}$ by (S.9).
2. M-step: Given $\gamma_{ik}^{(t)}$, update $\pi_k^{(t)}$, $\boldsymbol{\mu}_k^{(t)}$, and $\boldsymbol{\Theta}_k^{(t)}$ by (S.10), (S.11), and (S.12) respectively.

Until: $\sum_{k=1}^K \left\{ \frac{\left\|\boldsymbol{\mu}_k^{(t)} - \boldsymbol{\mu}_k^{(t-1)}\right\|_2}{\left\|\boldsymbol{\mu}_k^{(t-1)}\right\|_2} + \frac{\left\|\boldsymbol{\Theta}_k^{(t)} - \boldsymbol{\Theta}_k^{(t-1)}\right\|_F}{\left\|\boldsymbol{\Theta}_k^{(t-1)}\right\|_F} \right\}$ < a pre-specified cutoff (taken as 0.01 in our numerical study).
Return: The estimate of $\{\boldsymbol{\mu}_k^{(t)}, \boldsymbol{\Theta}_k^{(t)}, \pi_k^{(t)}, k = 1, \ldots, K\}$ at convergence.

C.1 Update of $\{\boldsymbol{\Theta}\}$ in the EM algorithm

Recall that maximizing (S.8) with respect to $\boldsymbol{\Theta}$ is equivalent to solving:

$$\{\boldsymbol{\Theta}_k^{(t)}, k = 1, \ldots, K\} = \underset{\{\boldsymbol{\Theta}\}}{\operatorname{argmax}} \left(\sum_{k=1}^K n_k \left[\log\{\det(\boldsymbol{\Theta}_k)\} - \operatorname{tr}\left(\widetilde{\boldsymbol{S}}_k \boldsymbol{\Theta}_k\right) \right] - \mathcal{P}(\{\boldsymbol{\Theta}\}) \right). \quad \text{(S.13)}$$

This can be efficiently achieved using the ADMM algorithm by modifying the joint graphical lasso algorithm in Danaher et al. (2014), summarized in Algorithm S2.

More specifically, this optimization can be reformulated as:

$$\underset{\{\boldsymbol{\Theta}, \boldsymbol{\Xi}\}}{\operatorname{argmin}} \left(-\sum_{k=1}^K n_k \left[\log\{\det(\boldsymbol{\Theta}_k)\} - \operatorname{tr}\left(\widetilde{\boldsymbol{S}}_k \boldsymbol{\Theta}_k\right) \right] + \mathcal{P}(\{\boldsymbol{\Xi}\}) \right), \quad \text{(S.14)}$$

subject to the constraint that $\boldsymbol{\Xi}_k = \boldsymbol{\Theta}_k, k = 1, \cdots, K$ as well as the positive definiteness con-

straint, where $\{\Xi\} = \Xi_1, \ldots, \Xi_K$, and $\Xi_k = (\xi_{kij})_{1 \leqslant i,j \leqslant p}$. The scaled augmented Lagrangian form for this problem is given by:

$$\begin{aligned}\mathcal{Q}_\kappa(\{\boldsymbol{\Theta}\}, \{\boldsymbol{\Xi}\}, \{\boldsymbol{\Psi}\}) = &- \sum_{k=1}^{K} n_k \left[\log\{\det(\boldsymbol{\Theta}_k)\} - \operatorname{tr}\left(\widetilde{\boldsymbol{S}}_k \boldsymbol{\Theta}_k\right)\right] + \mathcal{P}(\{\boldsymbol{\Xi}\}) \\ &+ \frac{\kappa}{2} \sum_{k=1}^{K} \|\boldsymbol{\Theta}_k - \boldsymbol{\Xi}_k + \boldsymbol{\Psi}_k\|_{\mathrm{F}}^2 - \frac{\kappa}{2} \sum_{k=1}^{K} \|\boldsymbol{\Psi}_k\|_{\mathrm{F}}^2,\end{aligned} \tag{S.15}$$

where $\{\boldsymbol{\Psi}\} = \boldsymbol{\Psi}_1, \ldots, \boldsymbol{\Psi}_K$ are dual variables, and κ is the penalty parameter. Throughout this study, we set $\kappa = 1$.

It is noted that the positive definiteness of the estimated precision matrices is naturally enforced by the update in Step 1 of Algorithm S2. The solution $\{\boldsymbol{\Theta}_1^{(t)}, \ldots, \boldsymbol{\Theta}_K^{(t)}\}$ for (S.13) obtained by Algorithm S2 is not necessarily symmetric in general. They can be symmetrized using the symmetrization strategy of Hao et al. (2018):

$$\theta_{kij}^{(t)} = \theta_{kij}^{(t)} I(|\theta_{kij}^{(t)}| \leqslant |\theta_{kji}^{(t)}|) + \theta_{kji}^{(t)} I(|\theta_{kij}^{(t)}| > |\theta_{kji}^{(t)}|). \tag{S.16}$$

Next, for solving (S.17), the efficient sparse alternating minimization algorithm (S-AMA, Wang et al. (2018)) can be used. Detailed iterative steps can be referred to Ren et al. (2022).

Algorithm S2 ADMM algorithm for solving (S.14)

Input: The pseudo sample covariance matrices $\widetilde{\boldsymbol{S}}_k, k = 1, \cdots, K$, tuning parameters λ_2', λ_3', and the penalty parameter κ.
Output: The estimated precision matrices $\{\boldsymbol{\Theta}_k, k = 1, \ldots, K\}$.
Initialization: $\boldsymbol{\Theta}_k^{(0)} = \boldsymbol{I}, \boldsymbol{\Xi}_k^{(0)} = \mathbf{0}, \boldsymbol{\Psi}_k^{(0)} = \mathbf{0}$, for $k = 1, \cdots, K$.
Repeat for $m = 1, 2, 3, \ldots$:

1. For $k = 1, \ldots, K$, update $\boldsymbol{\Theta}_k^{(m)}$ by solving

$$\underset{\{\boldsymbol{\Theta}\}}{\operatorname{argmin}} \left(-n_k \left[\log \{\det (\boldsymbol{\Theta}_k)\} - \operatorname{tr}\left(\widetilde{\boldsymbol{S}}_k \boldsymbol{\Theta}_k \right) \right] + \frac{\kappa}{2} \left\| \boldsymbol{\Theta}_k - \boldsymbol{\Xi}_k^{(m-1)} + \boldsymbol{\Psi}_k^{(m-1)} \right\|_{\mathrm{F}}^2 \right).$$

   Following Danaher et al. (2014), the solution is given by:

$$\boldsymbol{\Theta}_k^{(m)} = \boldsymbol{U} \widetilde{\boldsymbol{D}} \boldsymbol{U}^\top,$$

   where $\boldsymbol{U}\boldsymbol{D}\boldsymbol{U}^\top$ is the eigendecomposition of $\widetilde{\boldsymbol{S}}_k - \kappa \boldsymbol{\Xi}_k^{(m-1)}/n_k + \kappa \boldsymbol{\Psi}_k^{(m-1)}/n_k$, $\widetilde{\boldsymbol{D}}$ is a diagonal matrix with the jth diagonal element $\frac{n_k}{2\kappa}\left[-D_{jj} + \left(D_{jj}^2 + 4\kappa/n_k \right)^{1/2} \right]$, and D_{jj} is the jth diagonal element of $\boldsymbol{D}$.

2. Update $\{\boldsymbol{\Xi}^{(m)}\}$ by solving:

$$\underset{\{\boldsymbol{\Xi}\}}{\operatorname{argmin}} \left(\frac{\kappa}{2} \sum_{k=1}^K \|\boldsymbol{\Xi}_k - \boldsymbol{\mathcal{Z}}_k\|_{\mathrm{F}}^2 + \mathcal{P}(\{\boldsymbol{\Xi}\}) \right) \tag{S.17}$$

   using the S-AMA algorithm (Wang et al., 2018), where $\boldsymbol{\mathcal{Z}}_k = \boldsymbol{\Theta}_k^{(m)} + \boldsymbol{\Psi}_k^{(m-1)}$.

3. Update $\{\boldsymbol{\Psi}^{(m)}\}$ by $\boldsymbol{\Psi}_k^{(m)} = \boldsymbol{\Psi}_k^{(m-1)} + \boldsymbol{\Theta}_k^{(m)} - \boldsymbol{\Xi}_k^{(m)}$, for $k = 1, \ldots, K$.

Until: $\sum_{k=1}^K \frac{\left\| \boldsymbol{\Theta}_k^{(m)} - \boldsymbol{\Theta}_k^{(m-1)} \right\|_F}{\left\| \boldsymbol{\Theta}_k^{(m-1)} \right\|_F} < 10^{-2}$.
Return: The estimate of $\{\boldsymbol{\Theta}_k^{(m)}, k = 1, \ldots, K\}$ at convergence.

D Additional numerical results

D.1 Details of simulated settings in the main text

We consider a three-subgroup ($L = 3$) target domain with the number of variables $p = 100$ and the sample size $n_0 = 200$ across all subgroups. The target observations are generated as follows. First, the subgroup membership y_i's are randomly sampled from $\{1, 2, 3\}$. Then, $\boldsymbol{x}_i \sim N(\boldsymbol{\mu}(y_i), \boldsymbol{\Sigma}(y_i))$. The $(10y_i - 9)$-th to $(10y_i)$-th components of $\boldsymbol{\mu}(y_i)$ are 2, and the rest $p - 10$ components of the means are set as zero. For precision matrices, we simulate two types of network structures. For the first type, precision matrices of all subgroups are tridiagonal, where the diagonal elements are all equal to one, and the nonzero off-diagonal elements are $0.1I(y_i = 1) + 0.2I(y_i = 2) + 0.3I(y_i = 3)$. For the second type, the network of each subgroup consists of ten equally sized disjoint sub-networks, and we consider *power-law network* as the structure of each sub-network, in which power-law sub-networks are all generated with two edges added in each step. The initial ten-block precision matrix of the first subgroup $(\theta_{1ij})_{p\times p}$ is generated by $\theta_{1ij} = 1$, if $i = j$; $\theta_{1ij} = 0$, if $i \neq j, i \nsim j$; $\theta_{1ij} \sim$ Unif $([-0.5, -0.4] \cup [0.4, 0.5])$, if $i \neq j, i \sim j$, where $i \sim j$ means that there is an edge between nodes i and j, and $i \nsim j$ means otherwise. To ensure positive-definiteness, we set $\theta_{1jj} = \sum_{i\neq j} |\theta_{1ij}| + 0.1$. The precision matrices of second and third subgroups are generated following a similar mechanism, but they are all different from each other.

For auxiliary domains, we consider two different simulation examples. The objective of Example 1 is to demonstrate that the performance of the proposed approach improves with an increase in the number of informative auxiliary domains with overall similarity, while effectively filtering out interference from non-informative auxiliary domains. The objective of Example 2 is to demonstrate that the proposed method achieves robust local transfer in challenging situations where the informative auxiliary domain only has local similarity.

Example 1. (Overall similarity) We fix $K = 5$ and vary the number of informative

auxiliary domains $\text{card}(\mathcal{A}) \in \{0, 1, \cdots, K\}$. All auxiliary domains have the same subgroup number $L = 3$ as that of the target domain, and the sample size of all auxiliary subgroups is $3n_0$. For informative auxiliary domains, they have similar subgroup parameters to that of the target domain. Specifically, the informative auxiliary set of the l-th subgroup of the target domain $\mathcal{A}_l^{\Theta} = \mathcal{A}_l^{\mu} = \{(l', k) : l' = l, k = 1, \cdots, \text{card}(\mathcal{A})\}$, for $l = 1, 2, 3$, with $[\boldsymbol{\Delta}_{ll}^{(k)*}]_{(i,j)} = 0$ with probability 0.9 or randomly generated from $\text{Unif}[-0.1\sqrt{\frac{\log p}{n_0}}, 0.1\sqrt{\frac{\log p}{n_0}}]$ with probability 0.1, and $[\boldsymbol{\delta}_{ll}^{(k)*}]_j = 0$ with probability 0.9 or randomly generated from $\text{Unif}[-0.1\sqrt{\frac{\log p}{n_0}}, 0.1\sqrt{\frac{\log p}{n_0}}]$ with probability 0.1, for $i, j = 1, 2, \cdots, p$. For the l-th subgroup of non-informative auxiliary domains $k \in \{\text{card}(\mathcal{A}) + 1, \cdots, K\}$, $[\boldsymbol{\Delta}_{ll}^{(k)*}]_{(i,j)} = 0$ with probability 0.9 or randomly generated from $\text{Unif}[-10\sqrt{\frac{\log p}{n_0}}, 10\sqrt{\frac{\log p}{n_0}}]$ with probability 0.1, and $\boldsymbol{\mu}_l^{(k)*} = 2\boldsymbol{\mu}_l^*$.

Example 2. (Local similarity) We fix $K = 6$ and the number of subgroups for K auxiliary domains are $\{2, 3, 4, 3, 3, 3\}$ respecticely. The first three auxiliary domains are informative with local similarity, specifically, the k-th subgroup of the k-th informative auxiliary domain has similar parameters to the k-th subgroup of the target domain, for $k = 1, 2, 3$. Other auxiliary subgroups (whether in the first three informative or the last three non-informative auxiliary domains) are not similar to any target subgroup. The specific generation method of parameters and the sample size in auxiliary subgroups are similar to Example 1.

Table S2: Averaged metrics and their standard deviation in parenthesis for Example 1 under tridiagonal networks

Methods	card(A)	Per	L	CE	MSE-Mean	MSE-Precision	TPR	FPR
Proposed	0	0.84	3.3900(0.9309)	0.0130(0.0313)	0.1706(0.0896)	2.0709(1.1535)	0.8116(0.0297)	0.1779(0.0406)
	1	-	3.3900(0.9309)	0.0058(0.0159)	0.1317(0.0922)	0.9251(0.0720)	0.9928(0.0315)	0.0082(0.0028)
	2	-	3.3900(0.9309)	0.0070(0.0184)	0.1096(0.0974)	0.8801(0.0448)	0.9933(0.0315)	0.0002(0.0004)
	3	-	3.3900(0.9309)	0.0068(0.0181)	0.0988(0.0999)	0.8126(0.0523)	0.9928(0.0343)	0.0003(0.0001)
	4	-	3.3900(0.9309)	0.0067(0.0181)	0.0956(0.0988)	0.7840(0.0544)	0.9929(0.0339)	0.0001(0.0000)
	5	-	3.3900(0.9309)	0.0066(0.0177)	0.0959(0.0983)	0.7831(0.0540)	0.9929(0.0342)	0.0001(0.0001)
tlgmm	0	0	2.0000(0.0000)	0.2227(0.0004)	2.2074(0.0821)	4.1003(0.0727)	-	-
	1	-	2.0000(0.0000)	0.2227(0.0004)	2.2071(0.0823)	4.1004(0.0727)	-	-
	2	-	2.0000(0.0000)	0.2227(0.0004)	2.2062(0.0832)	4.1006(0.0727)	-	-
	3	-	2.0000(0.0000)	0.2226(0.0003)	2.2458(0.0089)	4.1724(0.0753)	-	-
	4	-	2.0000(0.0000)	0.2226(0.0000)	2.2424(0.0078)	4.1024(0.0726)	-	-
	5	-	2.0000(0.0000)	0.2226(0.0000)	2.2406(0.0078)	4.1030(0.0726)	-	-
HeteroGGM		0.84	3.3900(0.9309)	0.0130(0.0313)	0.1701(0.0878)	2.0709(1.1535)	0.8116(0.0297)	0.1779(0.0406)
SCAN		1	3.0000(0.0000)	0.0000(0.0000)	0.5365(0.0279)	1.2868(0.0994)	0.8068(0.1172)	0.0203(0.0034)
SCAN.v		0	2.0000(0.0000)	0.2226(0.0003)	2.3244(0.0316)	1.6102(0.0572)	0.7940(0.1203)	0.0280(0.0045)
JGL		0	2.0000(0.0000)	0.2226(0.0000)	2.2844(0.0229)	1.5106(0.0374)	0.8104(0.0579)	0.1038(0.0319)

Table S3: Averaged metrics and their standard deviation in parenthesis for Example 1 under block power-law networks

Methods	card(A)	Per	L	CE	MSE-Mean	MSE-Precision	TPR	FPR
Proposed	0	0.69	3.8700(1.3307)	0.0404(0.0624)	0.1775(0.0899)	3.5765(0.3082)	0.8658(0.0398)	0.1461(0.0443)
	1	-	3.8700(1.3307)	0.0052(0.0179)	0.1387(0.0942)	3.4177(0.4179)	0.8679(0.1748)	0.0136(0.0054)
	2	-	3.8700(1.3307)	0.0048(0.0169)	0.1117(0.1033)	3.0509(0.6440)	0.8698(0.1805)	0.0098(0.0032)
	3	-	3.8700(1.3307)	0.0047(0.0168)	0.1115(0.1029)	2.8289(0.7764)	0.8758(0.1808)	0.0073(0.0016)
	4	-	3.8700(1.3307)	0.0046(0.0160)	0.1056(0.1045)	2.6214(0.9146)	0.8777(0.1807)	0.0075(0.0020)
	5	-	3.8700(1.3307)	0.0044(0.0153)	0.1037(0.1044)	2.5746(0.9529)	0.8788(0.1816)	0.0065(0.0015)
tlgmm	0	0	2.0000(0.0000)	0.2227(0.0005)	2.1422(0.0379)	8.0246(0.1150)	-	-
	1	-	2.0000(0.0000)	0.2228(0.0005)	2.1415(0.0381)	8.0246(0.1150)	-	-
	2	-	2.0000(0.0000)	0.2228(0.0005)	2.1399(0.0383)	8.0247(0.1149)	-	-
	3	-	2.0000(0.0000)	0.2226(0.0000)	2.2395(0.0090)	7.9908(0.0611)	-	-
	4	-	2.0000(0.0000)	0.2226(0.0000)	2.2312(0.0079)	7.9934(0.0616)	-	-
	5	-	2.0000(0.0000)	0.2227(0.0005)	2.2398(0.0077)	8.0958(0.0603)	-	-
HeteroGGM		0.69	3.8700(1.3307)	0.0404(0.0624)	0.1771(0.0886)	3.5765(0.3082)	0.8658(0.0398)	0.1461(0.0443)
SCAN		1	3.0000(0.0000)	0.0000(0.0000)	0.6512(0.0412)	4.6900(0.0321)	0.8326(0.0128)	0.0341(0.0020)
SCAN.v		0	2.0000(0.0000)	0.2226(0.0000)	2.3806(0.0333)	6.7911(0.1226)	0.6464(0.0263)	0.0335(0.0017)
JGL		0	2.0000(0.0000)	0.2230(0.0010)	2.2764(0.0219)	5.7596(0.1455)	0.8229(0.0220)	0.1785(0.0086)

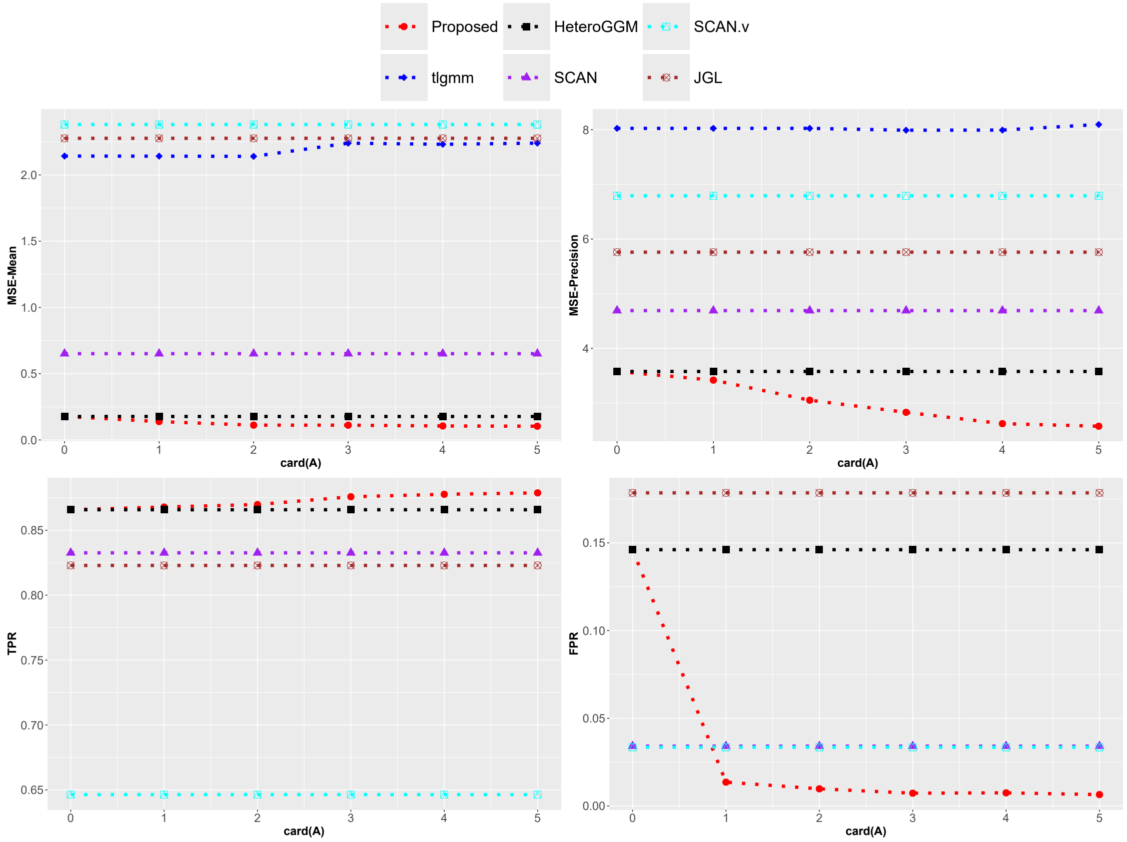

Figure S1: Averaged metrics of estimation errors over 100 replications for Example 1 with block power-law networks.

D.2 Biological interpretation of real data analysis

For the results in Subgroup 1, a subgroup benefiting from transfer learning, we first consider the commonalities before and after using LtransHeteroGGM. We focus on 8 hub genes with node degree both ranked among the top ten before and after information transfer, B2M, HLA-A, HLA-B, HLA-C, CD3D, CD3E, SLC25A5, and SLC25A6. It very interesting to note that these eight genes can be classified into three categories, where the proteins encoded by B2M, HLA-A, HLA-B, and HLA-C are components of MHC class I molecules, the proteins encoded by CD3D and CD3E are main components of T-cell receptor-CD3 complex, and the proteins encoded by SLC25A5 and SLC25A6 are main components of ADP/ATP translocase

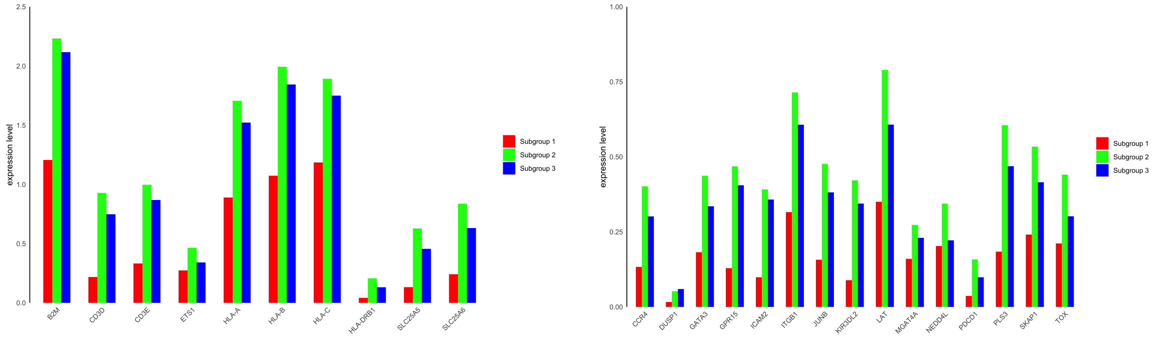

(a) (b)

Figure S2: (a) The expression levels of identified hub genes. (b) The average expression levels of marker genes.

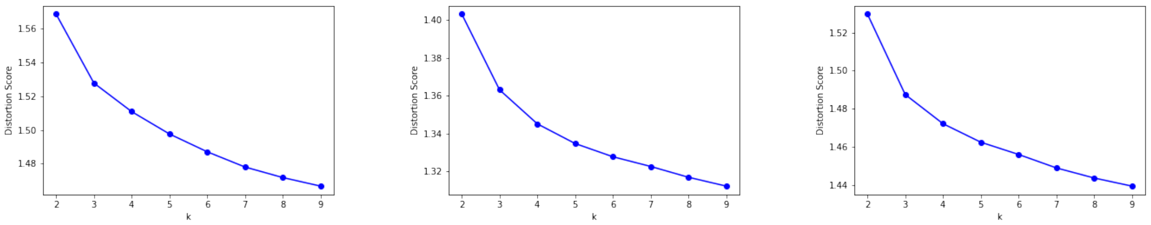

(a) (b) (c)

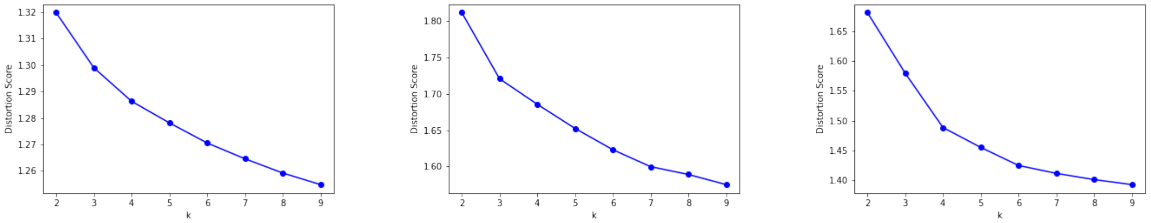

(d) (e) (f)

Figure S3: We apply "elbow method" to determine the upper bound of the number of subgroups, the result of "elbow method" suggests three subgroups in the target domain (subfigure a), and suggests three or four subgroups in auxiliary domains (subfigure b-f). Therefore, we set $M = 6$ in our analysis, which should be large enough.

(ANT). Some existing works have shown the activity of MHC class I molecules in CTCL. In SS, malignant T cells can suppress natural killer cell (NK)–mediated cytotoxic killing by upregulating surface class I MHC expression (Bouaziz et al., 2005), which is also true in MF (Chang et al., 2024). As a component of the T-cell receptor, T-cell receptor-CD3 complex is critical for T cell activation (Lanzavecchia et al., 1999). The activated T cells undergo clonal expansion and differentiation to perform their effector functions (Samelson, 2002). Besides, inappropriate T-cell activation via antigen presentation may be one of the pathogeneses of CTCL (Litvinov et al., 2016). ANT is a mitochondrial protein that facilitates the exchange of ADP and ATP across the mitochondrial inner membrane, plays an essential role in cellular energy metabolism. It is essential to the metabolic adaptation of cancer cells during tumoral development (Chevrollier et al., 2011). Moreover, the expression of ANT2, which has been shown to be growth-dependent, is considered as a marker of cell proliferation (Battini et al., 1987). Because of the role of these genes in malignant tumors, it is very natural for them to be hub nodes.

Notably, certain genes exhibit contrasting topological roles, functioning as hub nodes after information transfer, while transitioning to peripheral nodes before information transfer, such as HLA-DRB1 and ETS1, which demonstrate critical regulatory roles in Sézary syndrome pathogenesis. The HLA-DRB1 gene belongs to a group of MHC genes called MHC class II. MHC class II genes provide instructions for making proteins that are present on the surface of certain immune system cells. Woetmann et al. (2007) revealed their effect on the proliferation of the malignant CTCL cells. It is found that the malignant CTCL cells express MHC class II molecules, which are high-affinity receptors for bacterial superantigens such as staphylococcal enterotoxins (SE). SE triggers a bidirectional cross-talk between nonmalignant T cells and malignant CTCL cells that promotes the growth of the malignant cells. It is further shown in Woetmann et al. (2007) that nonmalignant T cells provide growth signals for malignant cells only when recognizing relevant MHC class II molecules on the malignant cells. Invasiveness

or metastatic potential is recognized as one of the primary hallmarks of cancer cells and is thought to play a role in tumor promotion (Hanahan and Weinberg, 2000). A large body of work has shown a role for ETS1 in regulating invasiveness and metastatic potential in a variety of cancer cell lines through the modulation of genes responsible for extracellular matrix remodeling, migration, and invasion (Seth and Watson, 2005; Hahne et al., 2005). It is also shown by experiments that ETS1 is required for tumor cell proliferation in vivo, and the inhibition of ETS1 blocks tumor formation (Holterman et al., 2010). Therefore, it is also a key gene in the formation of malignant cancer cells. These findings, to some extent, validate the capacity of transfer learning for identifying unique critical genes.

In addition to network structures, we further attempt to identify the cell types corresponding to these three subgroups. Some marker genes of SS have been widely reported in literature, including CCR4, DUSP1, GPR15, ICAM2, JUNB, KIR3DL2, PLS3, ITGB1, GATA3, NEDD4L, LAT, MGAT4A, PDCD1, SKAP1, and TOX, they should be highly expressed in SS cells. Their average expression levels in each subgroup are summarized in Figure S2(b) of the Supporting Information. It is noted that these genes are not in T-cell lymphoma pathway; as such, there is no overfitting problem. It is very interesting to note that the average expression levels of all marker genes in Subgroup 1 are significantly smaller that those in Subgroup 2, and the expression levels in Subgroup 3 were lying between Subgroup 1 and Subgroup 2 in magnitude, therefore, we speculate that cells in Subgroup 1, Subgroup 3, and Subgroup 2 may embody three consecutive stages of SS development. These marker genes with significant expression differences also exert indispensable regulatory effects on SS pathogenesis. For example, CCR4 plays a crucial role in the process of T cell extravasation to the skin (Ferenczi et al., 2002). TOX is aberrantly expressed in primary Sézary cells, correlating with the risk of disease-specific mortality, and its knockdown promotes apoptosis and reduces cell proliferation in CTCL cells (Huang et al., 2015).

Finally, we evaluate the stability of clustering of the proposed method and its competitors.

Specifically, we first remove 10 % samples randomly, and then apply each competing method. We calculate the proportion that the estimated subgroup is the same as that based on all the original samples. The stability of clustering is measured by clustering consistency (CC), defined as $CC = \binom{|S_{off}|}{2}^{-1} |\{(i,j) : I(\widehat{\varphi}(\boldsymbol{x}_i) = \widehat{\varphi}(\boldsymbol{x}_j)) = I(\varphi(\boldsymbol{x}_i) = \varphi(\boldsymbol{x}_j)); i < j, i, j \in S_{off}\}|$, where S_{off} denotes the set with 10 % samples off, $\widehat{\varphi}$ and φ are the estimated subgrouping memberships on data with 10 % samples off and original data, respectively. The process is repeated 50 times, and the CC of LtransHeteroGGM, heteroGGM, and SCAN are 0.978, 0.977, and 0.936, respectively. It is clear that the proposed method attains satisfactory stability performance.

E Additional simulation examples

E.1 Imbalanced subgroups

We consider an imbalanced three-subgroup ($L = 3$) target domain with the sample size $\{80, 160, 320\}$ for three subgroups, where the sample size of the subgroups doubled sequentially, and the total sample size (560) did not exceed the setting (600) in the main text. We consider a tridiagonal network structure. For the auxiliary domains, consider the local similarity of Example 2 to highlight the unique setting of this article. The metrics are summarized in Table S4.

For the single-task learning method based solely on the target domain, a slight performance degradation is observed compared to the balanced subgroup setting (e.g., for MSE-Precision of HeteroGGM: 2.9824 (imbalance) vs 2.0709 (balance)); nevertheless, the proposed method still delivers satisfactory performance (e.g., For MSE-Precision of LtransHeteroGGM: 1.2668 (imbalance) vs 1.2414 (balance)). This is attributed to effective local information transfer, which compensates for the performance decline induced by target domain imbalance. This demonstrates that the proposed method is not particularly sensitive to imbalanced settings.

Moreover, in the *imbalanced subgroup setting*, following the suggestion of a reviewer, we consider a new way for constructing indicators. Based on estimating the proportion of subgroups $\widehat{\pi}$, the indicators of different subgroups are weighted and summed according to their proportions, rather than simply averaging between subgroups. Such $\widehat{\pi}$-*weighted metrics* are presented in Table S5. Compared to the unweighted results (Table S4), all methods demonstrate improved performance with weighted metrics. This aligns with intuition: subgroups with smaller proportions tend to exhibit relatively poorer performance in parameter estimation. Assigning them lower weights effectively enhances the aggregate evaluation metrics. Notably, the proposed method consistently outperforms competing approaches, regardless of

whether metric weighting is applied.

Table S4: Averaged metrics and their standard deviation in parenthesis for Example 2 under nearest-neighbor networks

Methods	Per	L	CE	MSE-Mean	MSE-Precision	TPR	FPR
Proposed	0.93	3.2100(0.7693)	0.0157(0.0577)	0.1246(0.0714)	1.2668(0.0935)	0.7268(0.0694)	0.0447(0.0118)
tlgmm	0	2.0000(0.0000)	0.1106(0.0565)	1.6178(0.2767)	4.3729(0.0942)	-	-
HeteroGGM	0.93	3.2100(0.7693)	0.0171(0.0628)	0.1835(0.0579)	2.9824(0.6832)	0.7931(0.0395)	0.2041(0.0591)
SCAN	1	3.0000(0.0000)	0.0000(0.0000)	0.8236(0.0596)	1.5306(0.2122)	0.6930(0.3044)	0.0441(0.0196)
SCAN.v	0	2.0000(0.0000)	0.0818(0.0000)	2.6411(0.0365)	1.9565(0.0258)	0.8997(0.0165)	0.0260(0.0014)
JGL	0	2.0000(0.0000)	0.0995(0.0501)	2.4939(0.1743)	1.7915(0.1059)	0.7558(0.0459)	0.0963(0.0401)

Table S5: Averaged $\widehat{\pi}$-weighted metrics and their standard deviation in parenthesis for Example 2 under nearest-neighbor networks

Methods	Per	L	CE	MSE-Mean	MSE-Precision	TPR	FPR
Proposed	0.93	3.2100(0.7693)	0.0157(0.0577)	0.1139(0.0701)	1.2600(0.1229)	0.8361(0.0980)	0.0709(0.0196)
tlgmm	0	2.0000(0.0000)	0.1106(0.0565)	1.2513(0.2400)	3.5673(0.0750)	-	-
HeteroGGM	0.93	3.2100(0.7693)	0.0171(0.0628)	0.1620(0.0619)	2.2816(0.5576)	0.8935(0.0218)	0.1687(0.0627)
SCAN	1	3.0000(0.0000)	0.0000(0.0000)	0.6271(0.0449)	1.5851(0.3147)	0.7682(0.3371)	0.0326(0.0145)
SCAN.v	0	2.0000(0.0000)	0.0818(0.0000)	2.3370(0.1141)	1.9557(0.0264)	0.8998(0.0217)	0.0260(0.0024)
JGL	0	2.0000(0.0000)	0.0995(0.0501)	2.1539(0.0839)	1.4390(0.1056)	0.7769(0.0400)	0.0924(0.0387)

E.2 Comparisons for tlgmm

For the only transfer learning method suitable for comparison, tlgmm, which is an overall transfer limited by bivariate subgroups with identical covariance, low-dimensional data, and overall similarity between the target and the auxiliary domains, we further conducted a fair comparison under scenarios compatible with its assumptions, that is, there are two subgroups in all domains. Similar to Example 1, we consider the auxiliary domains with overall similarity, fix $K = 8$ and vary the number of informative auxiliary domains card($\mathcal{A}$) $\in \{0, 2, 4, 6, 8\}$. Moreover, we also consider the number of variables $p = 5$, with results summarized in Tables S6.

It is evident that due to the simplified setup, the clustering error metric approaches perfection. As for the MSEs for mean and precision matrix estimation, the following interesting conclusions are drawn:

Table S6: Averaged metrics and their standard deviation in parenthesis of HeteroGGM, tlgmm, and the proposed method for Example 1 under block tridiagonal networks with $p = 5$.

Methods	card(A)	Per	L	CE	MSE-Mean	MSE-Precision	TPR	FPR
Proposed	0	1	2.0000(0.0000)	0.0000(0.0005)	0.0713(0.0269)	0.1275(0.0062)	1.0000(0.0000)	0.0000(0.0000)
	2	1	2.0000(0.0000)	0.0000(0.0005)	0.0497(0.0366)	0.0977(0.0042)	1.0000(0.0000)	0.0000(0.0000)
	4	1	2.0000(0.0000)	0.0000(0.0005)	0.0434(0.0361)	0.0887(0.0038)	1.0000(0.0000)	0.0000(0.0000)
	6	1	2.0000(0.0000)	0.0000(0.0005)	0.0396(0.0328)	0.0700(0.0057)	1.0000(0.0000)	0.0000(0.0000)
	8	1	2.0000(0.0000)	0.0000(0.0005)	0.0380(0.0291)	0.0604(0.0062)	1.0000(0.0000)	0.0000(0.0000)
tlgmm	0	1	2.0000(0.0000)	0.0000(0.0005)	0.1527(0.0307)	0.1940(0.0444)	-	-
	2	1	2.0000(0.0000)	0.0000(0.0005)	0.1529(0.0305)	0.1940(0.0444)	-	-
	4	1	2.0000(0.0000)	0.0000(0.0005)	0.1098(0.0295)	0.1947(0.0444)	-	-
	6	1	2.0000(0.0000)	0.0000(0.0005)	0.0342(0.0005)	0.1942(0.0438)	-	-
	8	1	2.0000(0.0000)	0.0000(0.0005)	**0.0249**(0.0013)	0.1942(0.0437)	-	-
HeteroGGM		1	2.0000(0.0000)	0.0000(0.0005)	0.0714(0.0269)	0.2409(0.0366)	0.9712(0.0685)	0.3458(0.1878)

- Regarding the MSE for the mean: The performance of the tlgmm method improves progressively as the number of informative auxiliary domains increases. *When all auxiliary domains are informative and globally similar to the target domain, tlgmm achieves its best performance, even slightly outperforming our approach.* However, when the number of informative auxiliary domains is limited, the tlgmm method performs worse than the target-domain-only approach. This reveals a limitation of its overall transfer mechanism, where negative transfer is difficult to avoid.

- Regarding the MSE for the precision matrix: Even with a sufficient number of informative auxiliary domains, the tlgmm method still exhibits suboptimal performance. This is likely attributable to its failure to account for sparsity.

Overall, these conclusions align well with the intuition behind their method design.

E.3 More subnetwork structures

We further consider a block nearest-neighbor network, in which the network consists of ten equally sized disjoint subnetworks (modules). Among the ten subnetworks, eight are shared by the three subgroups. In addition, subgroups 1 and 2, as well as subgroups 1 and 3 share one subnetwork. Subgroups 2 and 3 also have a unique subnetwork of its own.

As for nearest-neighbor network structures, in each subnetwork, we generate $p/10$ points randomly on a unit square, calculate all $p/10 \times (p/10 - 1)/2$ pairwise distances, and find the m nearest neighbors of each point. The nearest-neighbor network is obtained by linking any two points that are among the m-nearest neighbors of each other. The integer m controls the degree of sparsity, and we set $m = 2$ in our simulation. The results under Examples 1 and 2 are respectively summarized in Tables S7 and S8. Observations made are very similar to those under the existing settings.

Table S7: Averaged metrics and their standard deviation in parenthesis for Example 1 under nearest-neighbor networks

Methods	card(A)	Per	L	CE	MSE-Mean	MSE-Precision	TPR	FPR
Proposed	0	0.64	3.48(0.7124)	0.0267(0.0393)	0.2285(0.1685)	1.9268(0.1537)	0.9789(0.0182)	0.1434(0.0358)
	1	-	3.48(0.7124)	0.0209(0.0325)	0.1972(0.1771)	1.8276(0.0767)	0.9921(0.0088)	0.0117(0.0035)
	2	-	3.48(0.7124)	0.0199(0.0319)	0.1825(0.1828)	1.7482(0.0985)	0.9999(0.0004)	0.0067(0.0010)
	3	-	3.48(0.7124)	0.0191(0.0299)	0.1723(0.1864)	1.5380(0.0893)	0.9998(0.0011)	0.0053(0.0008)
	4	-	3.48(0.7124)	0.0185(0.0293)	0.1699(0.1869)	1.3673(0.1080)	0.9994(0.0035)	0.0046(0.0005)
	5	-	3.48(0.7124)	0.0177(0.0315)	0.1692(0.1869)	1.3444(0.1003)	1.0000(0.0003)	0.0035(0.0004)
tlgmm	0	0	2.00(0.0000)	0.2230(0.0007)	2.1754(0.0805)	6.3921(0.0568)	-	-
	1	0	2.00(0.0000)	0.2230(0.0007)	2.1746(0.0808)	6.3921(0.0569)	-	-
	2	0	2.00(0.0000)	0.2231(0.0008)	2.1716(0.0818)	6.3919(0.0567)	-	-
	3	0	2.00(0.0000)	0.2229(0.0010)	2.2592(0.0124)	6.3654(0.0396)	-	-
	4	0	2.00(0.0000)	0.2229(0.0010)	2.2472(0.0120)	6.3655(0.0392)	-	-
	5	0	2.00(0.0000)	0.2229(0.0010)	2.2444(0.0120)	6.3654(0.0392)	-	-
HeteroGGM		0.64	3.48(0.7124)	0.0267(0.0393)	0.2342(0.1711)	1.9268(0.1537)	0.9789(0.0182)	0.1434(0.0358)
SCAN		1	3.00(0.0000)	0.0000(0.0000)	0.7411(0.0481)	3.1142(0.0273)	0.9587(0.0077)	0.0874(0.0030)
SCAN.v		0	2.00(0.0000)	0.2226(0.0000)	2.4278(0.0546)	5.0750(0.0826)	0.8490(0.0202)	0.0709(0.0035)
JGL		0	2.00(0.0000)	0.2234(0.0017)	2.3016(0.0373)	4.4427(0.0450)	0.8989(0.0139)	0.1826(0.0035)

Table S8: Averaged metrics and their standard deviation in parenthesis for Example 2 under nearest-neighbor networks

Methods	Per	L	CE	MSE-Mean	MSE-Precision	TPR	FPR
Proposed	0.64	3.48(0.7124)	0.0221(0.0354)	0.1860(0.1645)	1.8837(0.4675)	0.9747(0.0783)	0.0079(0.0025)
tlgmm	0	2.00(0.0000)	0.2230(0.0009)	2.2369(0.1118)	6.3960(0.0551)	-	-
HeteroGGM	0.64	3.48(0.7124)	0.0267(0.0393)	0.2342(0.1711)	1.9268(0.1537)	0.9789(0.0182)	0.1434(0.0358)
SCAN	1	3.00(0.0000)	0.0000(0.0000)	0.7411(0.0481)	3.1142(0.0273)	0.9587(0.0077)	0.0874(0.0030)
SCAN.v	0	2.00(0.0000)	0.2226(0.0000)	2.4278(0.0546)	5.0750(0.0826)	0.8490(0.0202)	0.0709(0.0035)
JGL	0	2.00(0.0000)	0.2234(0.0017)	2.3016(0.0373)	4.4427(0.0450)	0.8989(0.0139)	0.1826(0.0035)

E.4 Large discrepancies in subgroup numbers between target and auxiliary domains

In fact, our proposed method does not require the informative auxiliary domain(s) near-matching the target domain overall. It only necessitates that individual subgroups within the auxiliary domain exhibit parameter similarity with certain subgroups in the target domain. The remaining subgroups within that auxiliary domain can be highly dissimilar to any subgroup in the target domain (as visually demonstrated in Figure 1 of the main text). Furthermore, for informative auxiliary domains and the target domain, our method does not require either a close match in the number of subgroups or a near-matching subgroup structure.

In our existing Simulation Example 2, the target domain had 3 subgroups, and the auxiliary domains never exceeded 4 subgroups. This might have inadvertently suggested a requirement for nearly identical subgroup counts. To clarify this point, we have added a new simulation case. Keeping the target domain unchanged (3 subgroups), we now consider three auxiliary domains exhibiting local similarity, each configured with 9 subgroups – significantly exceeding the target domain's count of 3. Similar to the original Example 2, the k-th subgroup of the k-th informative auxiliary domain has similar parameters to the k-th subgroup of the target domain, for $k = 1, 2, 3$. Crucially, this means each auxiliary domain contains 8 completely non-informative interfering auxiliary subgroups, with only one informative subgroup. This scenario simulates conditions where:

- The number of subgroups differs substantially between auxiliary and target domains.
- The subgroup structure of the auxiliary domains is more complex.

The results, presented in Table S9, demonstrate that the performance of our proposed method remains satisfactory. It significantly outperforms all competing methods and is not

adversely affected by the presence of these more complex auxiliary domains with numerous non-informative subgroups.

Table S9: Averaged metrics and their standard deviation in parenthesis for Example 2 under block tridiagonal networks and large discrepancies in subgroup numbers between target and auxiliary domains.

Methods	Per	L	CE	MSE-Mean	MSE-Precision	TPR	FPR
Proposed	0.88	3.30(0.8472)	0.0122(0.0340)	0.1167(0.0939)	1.2246(0.0804)	0.8553(0.1267)	0.0359(0.0099)
tlgmm	0	2.00(0.0000)	0.2226(0.0002)	2.3032(0.0411)	4.0979(0.0651)	-	-
HeteroGGM	0.88	3.30(0.8472)	0.0134(0.0375)	0.1642(0.0837)	1.8088(0.6199)	0.8211(0.0207)	0.1775(0.0352)
SCAN	1	3.00(0.0000)	0.0000(0.0000)	0.5349(0.0257)	1.2712(0.0261)	0.8243(0.0194)	0.0204(0.0020)
SCAN.v	0	2.00(0.0000)	0.2226(0.0000)	2.3257(0.0305)	1.6090(0.0572)	0.7958(0.1191)	0.0280(0.0045)
JGL	0	2.00(0.0000)	0.2226(0.0000)	2.2823(0.0232)	1.5099(0.0373)	0.8066(0.0572)	0.0989(0.0322)

E.5 The effect of the hard-threshold cutoff

To investigate whether the cutoff-based approach maintains robust performance under scenarios involving increasingly complex auxiliary domains, we designed a new simulation study. In this simulation, the structural complexity of the auxiliary domains is progressively increased. Specifically, analogous to Example 2 in the main text, there are still three informative auxiliary domains with local similarity, with subgroup numbers of 2, 3, and 4, respectively. However, both the number of auxiliary domains and the number of internal subgroups are increased, that is, the numbers of subgroups for K auxiliary domains are $\{2,3,4,2,3,4,5,6\}$ ($K = 8$), $\{2,3,4,2,3,4,5,6,7,8\}$ ($K = 10$), and $\{2,3,4,2,3,4,5,6,7,8,9,10\}$ ($K = 12$), respectively.

The results, presented in Tables S10, demonstrate that despite the gradational increase in both the number of non-informative auxiliary domains and the complexity of their internal subgroup structures, the performance of the cutoff-based method exhibits no degradation.

Table S10: Averaged metrics and their standard deviation in parenthesis for the example where the number of non-informative auxiliary domains and the number of internal non-informative subgroups are increased

Methods	card(A)	Per	L	CE	MSE-Mean	MSE-Precision	TPR	FPR
Proposed	8	0.87	3.62(1.1469)	0.0055(0.0220)	0.1287(0.0745)	1.2165(0.0696)	0.8809(0.1062)	0.0379(0.0077)
Proposed	10	0.87	3.62(1.1469)	0.0074(0.0310)	0.1346(0.1075)	1.2548(0.1060)	0.8132(0.1682)	0.0325(0.0134)
Proposed	12	0.87	3.62(1.1469)	0.0076(0.0280)	0.1404(0.1096)	1.2589(0.1066)	0.8006(0.1721)	0.0319(0.0139)
tlgmm	8	0	2.00(0.0000)	0.2313(0.0463)	2.2297(0.1499)	4.1899(0.1903)	-	-
tlgmm	10	0	2.00(0.0000)	0.2228(0.0007)	2.2440(0.0630)	4.1379(0.0947)	-	-
tlgmm	12	0	2.00(0.0000)	0.2486(0.0658)	2.2600(0.2010)	4.3237(0.3865)	-	-
HeteroGGM		0.87	3.62(1.1469)	0.0191(0.0496)	0.1808(0.0966)	2.1050(0.9127)	0.8168(0.0232)	0.1952(0.0577)
SCAN		1	3.00(0.0000)	0.0000(0.0000)	0.5353(0.0220)	1.2914(0.1254)	0.7892(0.1527)	0.0203(0.0042)
SCAN.v		0	2.00(0.0000)	0.2227(0.0006)	2.3265(0.0359)	1.6060(0.0608)	0.7745(0.1298)	0.0276(0.0046)
JGL		0	2.00(0.0000)	0.2226(0.0000)	2.2872(0.0252)	1.5001(0.0340)	0.8138(0.0659)	0.1098(0.0307)

E.6 Simulations under the misspecified subgroup number

In reality, the number of target subgroups may indeed be identified incorrectly, leading to poor initialization. Although the proposed method exhibits a moderate dependence on initialization, especially the correct number of subgroups, the safeguard against negative transfer inherent in the proposed framework ensures that initial bias is not amplified by the local transfer mechanism, even under poor initialization. This safety arises because significant distributional shifts in poorly initialized target subgroups (including the wrong subgroup number) relative to the true distribution typically result in the absence of closely matching auxiliary subgroups. Consequently, during the pre-screening step of the framework, the informative auxiliary set can be identified as an empty set, leading to the immediate termination of information transfer. This mechanism effectively prevents the amplification of initial estimation errors.

To verify this, we design a new simulation. When executing all competing methods, we deliberately specified an incorrect number of subgroups (true value = 3; enforced settings = 2, 4, 5), with results in Tables S11 - S13. **Although the performance of all methods degraded under this misspecification, the proposed method still exhibited some improvement over target-domain-only approaches**. The underlying reason may be as follows: Even though the erroneous subgroup count causes the initial subgroup assignments

to deviate substantially from the true distribution, some subgroups might still be identified reasonably well. For instance, when enforcing 2 subgroups, the true three subgroups might be forced into a configuration where the first two are incorrectly merged, but the third subgroup remains correctly identified; when enforcing 4 subgroups, the true three subgroups might be split such that the first subgroup is incorrectly fragmented, but the latter two remain correctly identified. For subgroups that are correctly initialized, the proposed local transfer approach can leverage relevant informative auxiliary subgroups to improve their estimation accuracy. Conversely, for subgroups suffering from severe initialization errors, during the pre-screening step of the framework, the informative auxiliary set can be identified as an empty set, leading to the immediate termination of information transfer, so that applying the localized transfer method does not lead to further degradation.

Therefore, the overall conclusion is: If the initialized subgroup structure is entirely disrupted (including the wrong subgroup number), the proposed localized transfer method does not amplify the existing bias. If the initialized structure retains partially correct assignments (even if the subgroup number is wrong), the proposed localized transfer method can still enhance estimation for these correctly identified subgroups.

Table S11: Averaged metrics and their standard deviation in parenthesis for Example 2 under block tridiagonal networks and misspecified subgroup number ($L = 2$).

Methods	Per	L	CE	MSE-Mean	MSE-Precision	TPR	FPR
Proposed	0	2.0000(0.0000)	0.2226(0.0000)	2.2266(0.1322)	1.6297(0.2271)	0.9388(0.0328)	0.0966(0.0057)
tlgmm	0	2.0000(0.0000)	0.2226(0.0000)	2.1682(0.0660)	4.1787(0.0813)	-	-
HeteroGGM	0	2.0000(0.0000)	0.2226(0.0000)	2.2036(0.1237)	2.1687(0.2147)	0.8538(0.0828)	0.1340(0.0288)
SCAN.v	0	2.0000(0.0000)	0.2226(0.0000)	2.3257(0.0305)	1.9090(0.0572)	0.7958(0.1191)	0.0280(0.0045)
JGL	0	2.0000(0.0000)	0.2226(0.0000)	2.2823(0.0232)	1.8099(0.0373)	0.8066(0.0572)	0.0989(0.0322)

*It should be noted that when the estimated number of subgroups diverges from the true number, the estimation error for the subgroup parameters is calculated following the established convention from Ren et al. (2022). Specifically, each estimated subgroup is matched to the closest true subgroup based on parameter similarity, and the error is computed via this matching procedure. Consequently, under subgroup number misspecification, the reported estimation error metric, while exhibiting some degradation compared to the scenario with the correct subgroup count, is not severely compromised.

Table S12: Averaged metrics and their standard deviation in parenthesis for Example 2 under block tridiagonal networks and misspecified subgroup number ($L = 4$).

Methods	Per	L	CE	MSE-Mean	MSE-Precision	TPR	FPR
Proposed	0	4.0000(0.0000)	0.0359(0.0179)	0.2540(0.1139)	1.4883(0.1355)	0.7602(0.0774)	0.0201(0.0014)
tlgmm	0	2.0000(0.0000)	0.2226(0.0000)	2.1682(0.0660)	4.1787(0.0813)	-	-
HeteroGGM	0	4.0000(0.0000)	0.0550(0.0009)	0.2879(0.0987)	2.7471(1.0013)	0.8486(0.0548)	0.2430(0.1038)
SCAN.v	0	4.0000(0.0000)	0.0132(0.0184)	1.7858(0.4746)	2.2686(2.2091)	0.0978(0.2363)	0.0043(0.0114)
JGL	0	4.0000(0.0000)	0.0548(0.0012)	0.7369(0.0145)	1.3877(0.0271)	0.6693(0.0194)	0.0500(0.0035)

*The tlgmm forces consideration of two subgroups and will not change with our specification.

Table S13: Averaged metrics and their standard deviation in parenthesis for Example 2 under block tridiagonal networks and misspecified subgroup number ($L = 5$).

Methods	Per	L	CE	MSE-Mean	MSE-Precision	TPR	FPR
Proposed	0	5.0000(0.0000)	0.0656(0.0211)	0.3124(0.1254)	1.4236(0.1357)	0.6011(0.0880)	0.0123(0.0023)
tlgmm	0	2.0000(0.0000)	0.2226(0.0000)	2.1611(0.0615)	4.1803(0.0889)	-	-
HeteroGGM	0	5.0000(0.0000)	0.0916(0.0184)	0.3427(0.1099)	3.1695(0.8476)	0.8273(0.0363)	0.2466(0.0492)
SCAN.v	0	5.0000(0.0000)	0.0406(0.0520)	1.9746(0.3415)	2.2375(1.3730)	0.0657(0.1311)	0.0087(0.0184)
JGL	0	5.0000(0.0000)	0.1084(0.0074)	0.7591(0.0316)	1.3224(0.0407)	0.5481(0.0326)	0.0323(0.0108)

*The tlgmm forces consideration of two subgroups and will not change with our specification.

F Additional real data analysis results

F.1 Network structure comparison

Table S14: The network characteristics of Subgroup 1 before and after transfer. (A hub node here is defined as a node with a degree exceeding 20.)

	network before transferred	network after transferred
number of edges	245	382
density	0.13	0.20
hub nodes	**B2M HLA-B HLA-A CD3E HLA-C SLC25A6** HLA-E **CD3D**	HLA-DRB1 **HLA-A B2M** ETS1 SLC25A5 **HLA-B SLC25A6 CD3E CD3D HLA-C** JAK1

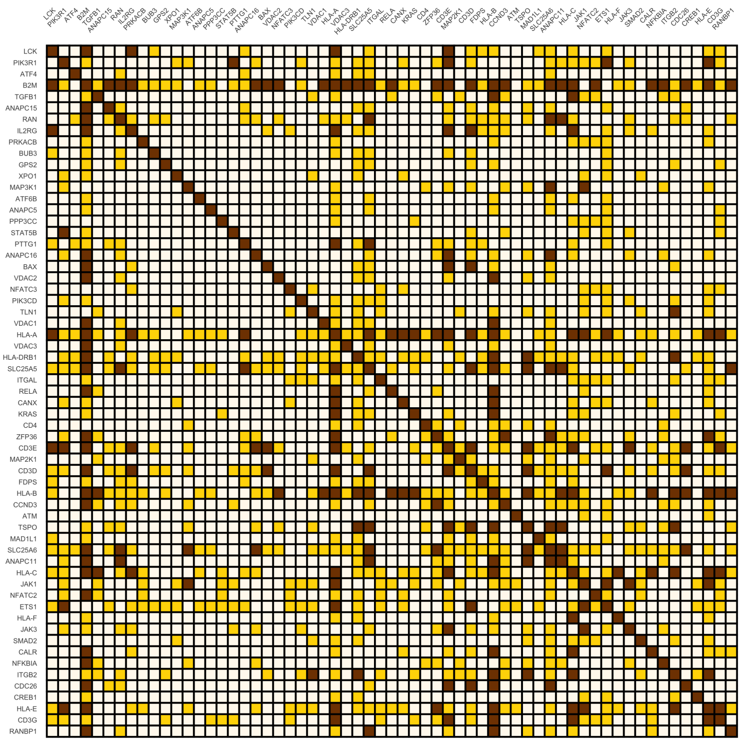

Figure S4: The heatmap of the type of edges in Subgroup 1 before and after transfer. The brown color indicates the edge is shared before and after transfer, the gold color indicates the edge is only connected before or after transfer, and the white color indicates the edge does not exist before and after transfer.

F.2 Solution paths

Figure S5 and Figure S6 show the solution paths of the number of subgroups and the F-norm of the parameters in different subgroups against λ, where λ is the parameter controlling the number of subgroups. To better present the solution paths, λ should take densely spaced values in a smaller interval, so the X-axis is displayed on a $\log(\lambda)$ scale. The results show that as λ increases, samples in nearby subgroups merge gradually, eventually forming a single group.

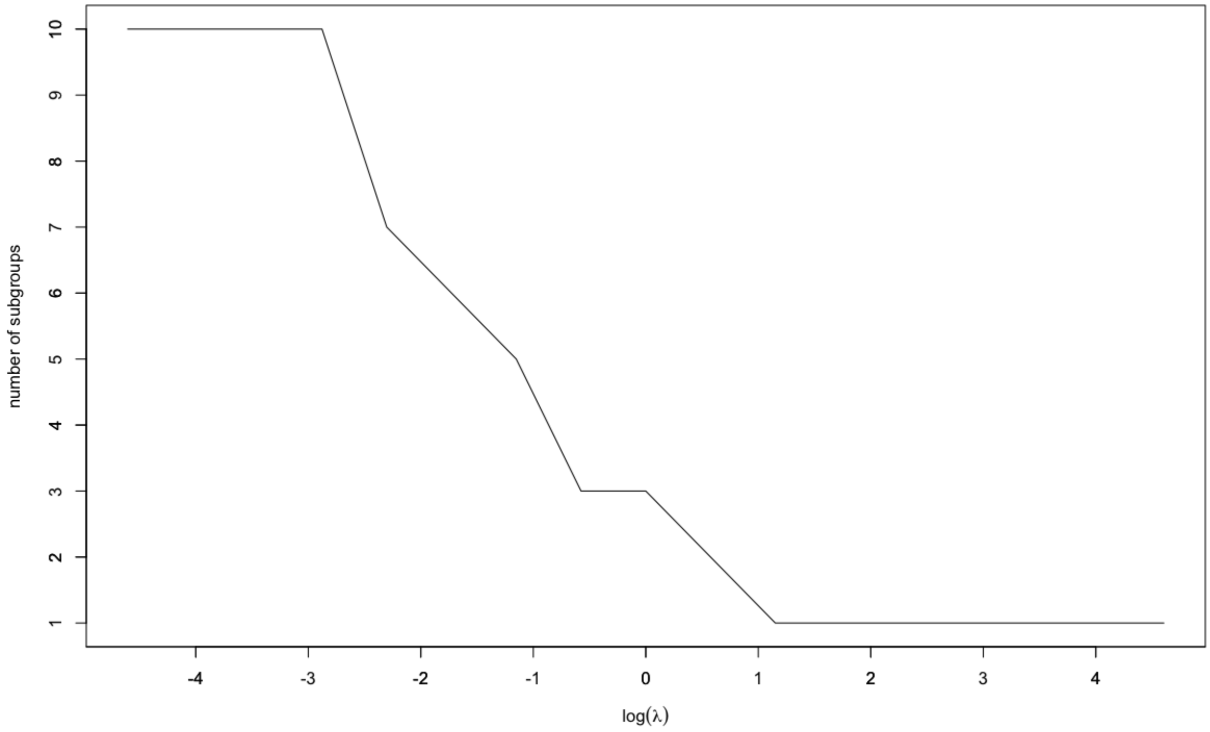

Figure S5: Solution paths for the number of subgroups against $\log(\lambda)$.

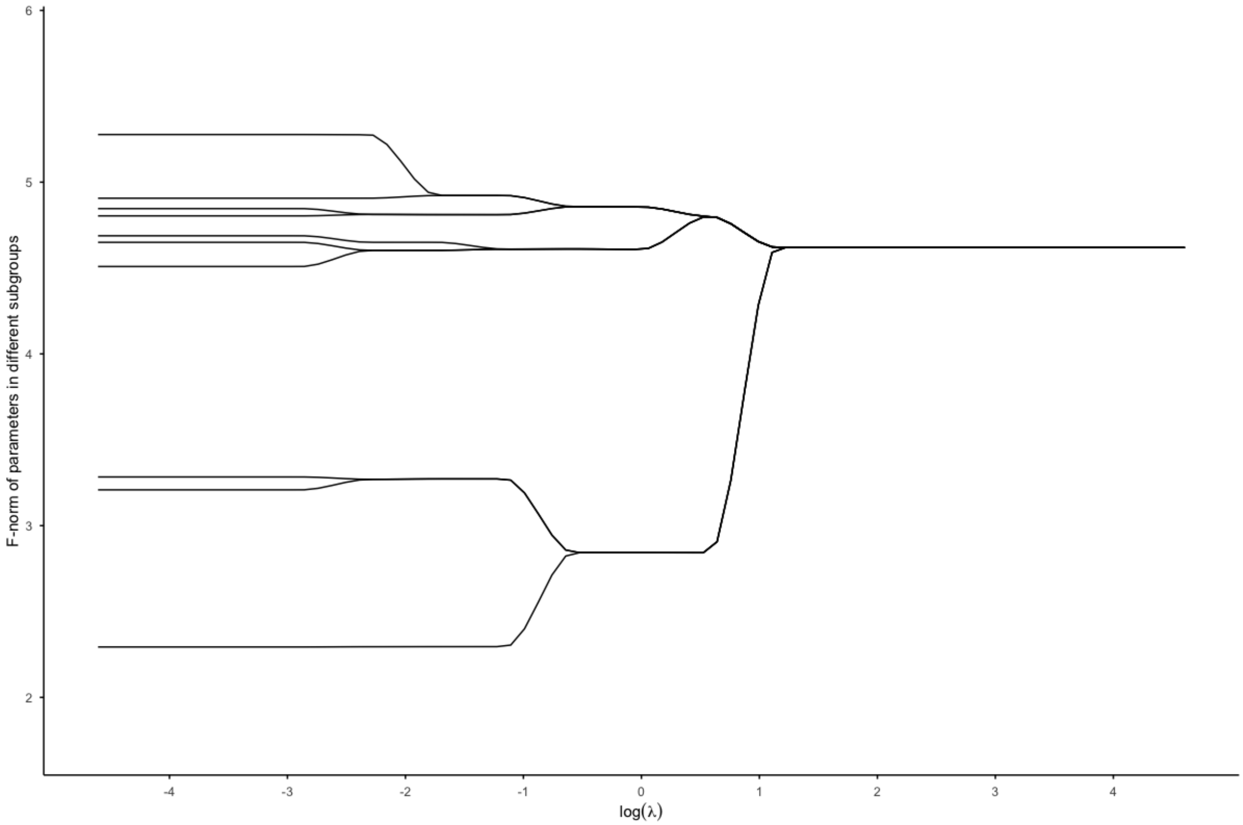

Figure S6: Solution paths for the F-norm of the parameters in different subgroups against $\log(\lambda)$.

F.3 Results under other numbers of subgroups

In this section, we present the results under the number of subgroups, 2, 4, and 5, respectively, and then we discuss how the performance of the proposed method will be affected. For the convenience of notation, when the number of subgroups is L, the subgroup indices are denoted as $1^{(L)}, \cdots, L^{(L)}$, respectively.

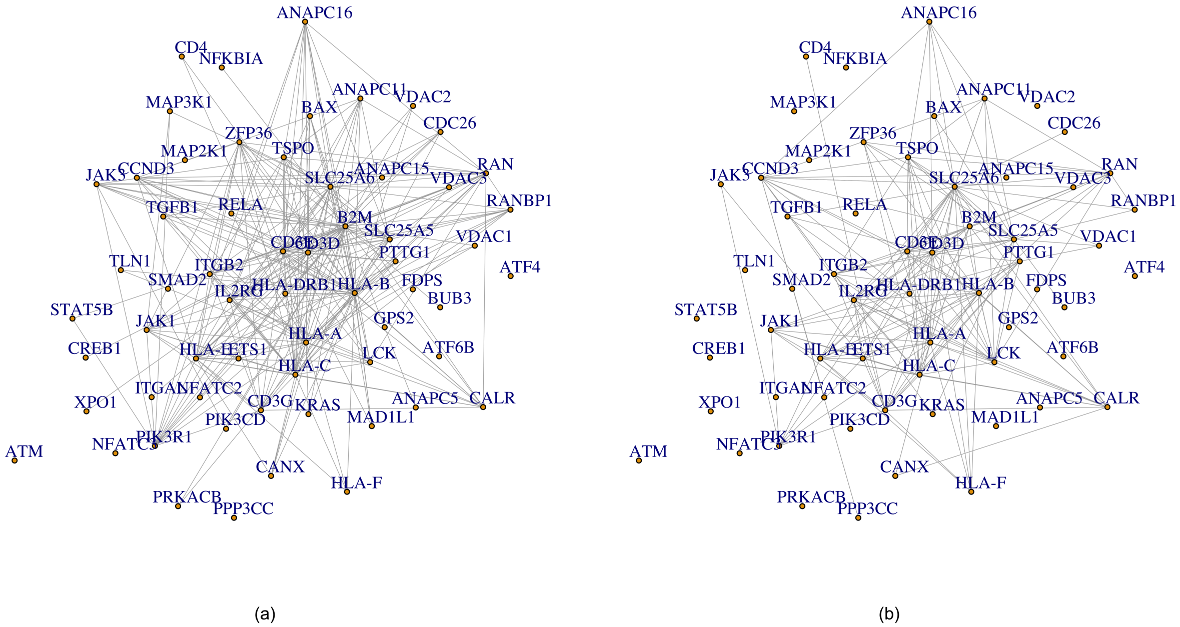

Figure S7: The network structures for 2 subgroups. (a)-(b): the network structures of $1^{(2)}$ and $2^{(2)}$, respectively.

We first analyze the differences in network structures for $L = 2$ and $L = 3$, the network structures for $L = 2$ are plotted in Figure S7. By tracing the sample indices, we find that the samples in $3^{(3)}$ split into two parts, where the vast majority of them and samples from $2^{(3)}$ have merged to form $2^{(2)}$, a small part of them and samples from $1^{(3)}$ have merged to form $1^{(2)}$, and there is no informative auxiliary domain. The network structure of $1^{(2)}$ is similar to the structure of $1^{(3)}$ before transfer, and the heatmap of the type of edges in $1^{(2)}$ and $1^{(3)}$ before transfer is shown in Figure S8.

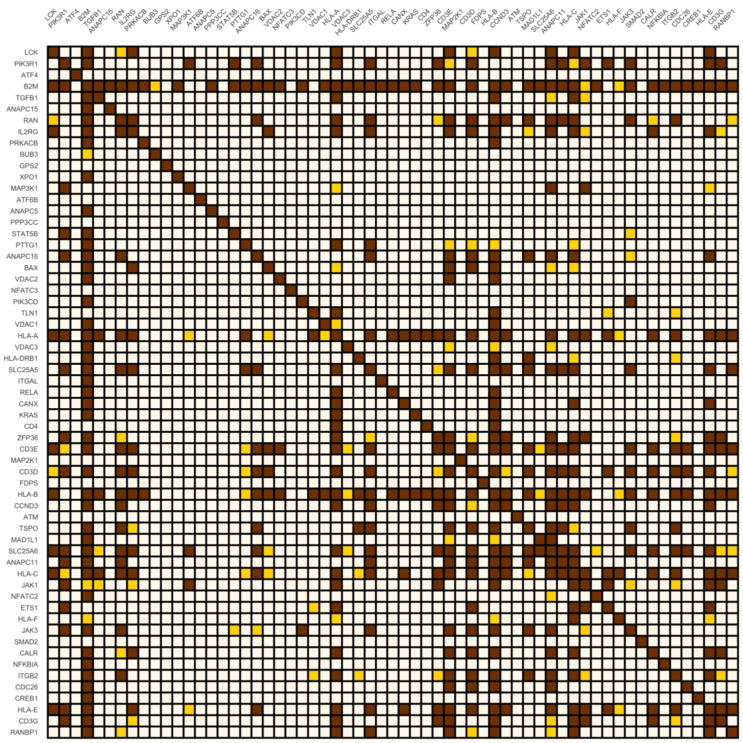

Figure S8: The heatmap illustrating the differences in edges between $1^{(2)}$ and $1^{(3)}$ before transfer. The brown color indicates the edge is shared in two networks, the gold color indicates the edge is only connected in $1^{(2)}$ or $1^{(3)}$ before transfer, and the white color indicates the edge does not exist in either of the two networks.

Next, we focus on the differences in network structures for $L = 4$ and $L = 3$. The network structures for $L = 4$ are given in Figure S9. By tracing the sample indices, we find that the vast majority of samples from $1^{(4)}$ form $1^{(3)}$, the vast majority of samples from $2^{(4)}$ and $3^{(4)}$ have merged to form $2^{(3)}$, the vast majority of samples from $4^{(4)}$ form $3^{(3)}$, and the learning parameters in $1^{(4)}$ are transferred. The heatmap of the type of edges in $1^{(4)}$ and $1^{(3)}$, and the heatmap of the type of edges in $4^{(4)}$ and $3^{(3)}$ are shown in Figure S10 and S11.

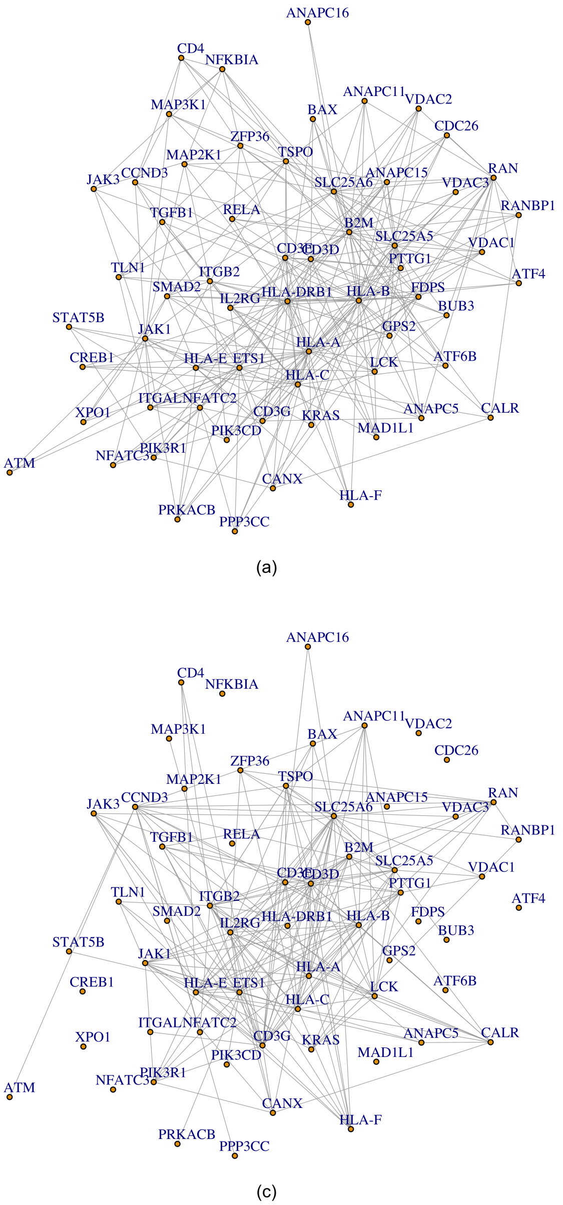

Figure S9: The network structure for 4 subgroups. (a)-(d): the network structures of $1^{(4)}$, $2^{(4)}$, $3^{(4)}$ and $4^{(4)}$, respectively.

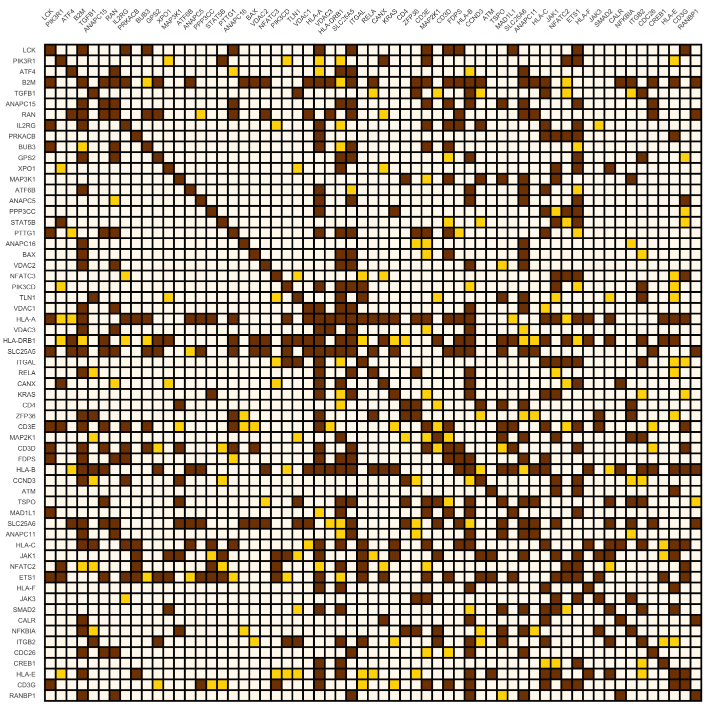

Figure S10: The heatmap illustrating the differences in edges between $1^{(4)}$ and $1^{(3)}$.

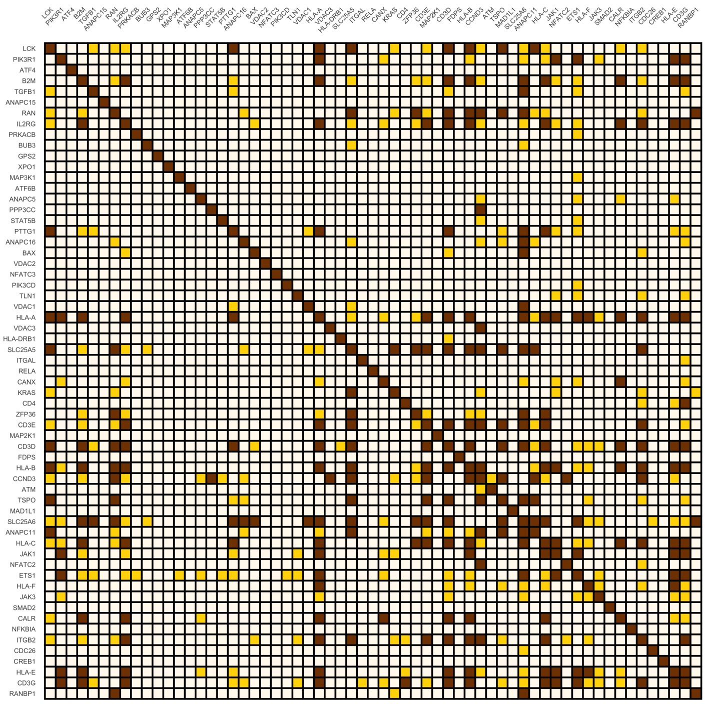

Figure S11: The heatmap illustrating the differences in edges between $4^{(4)}$ and $3^{(3)}$.

Finally, we discuss how the performance of the proposed method will be affected when $L = 5$. The network structures for $L = 5$ are shown in Figure S12. By tracing the sample indices, we find that the vast majority of samples from $1^{(5)}$ and $2^{(5)}$ have merged to form $1^{(4)}$, the vast majority of samples from $3^{(5)}$ form $2^{(4)}$, the vast majority of samples from $4^{(5)}$ form $3^{(4)}$, the vast majority of samples from $5^{(5)}$ form $4^{(4)}$, and there is no informative auxiliary domain. The network structure of $5^{(5)}$ is similar to the structure of $3^{(3)}$, and the heatmap illustrating the differences in edges between $5^{(5)}$ and $3^{(3)}$ is shown in Figure S13.

For a better understanding of the relationships between subgroups corresponding to different L, we plot the merged trajectories of the subgroups; only those trajectories that are representative of the majority of samples in each subgroup are considered. The trajectories are shown in Figure S14.

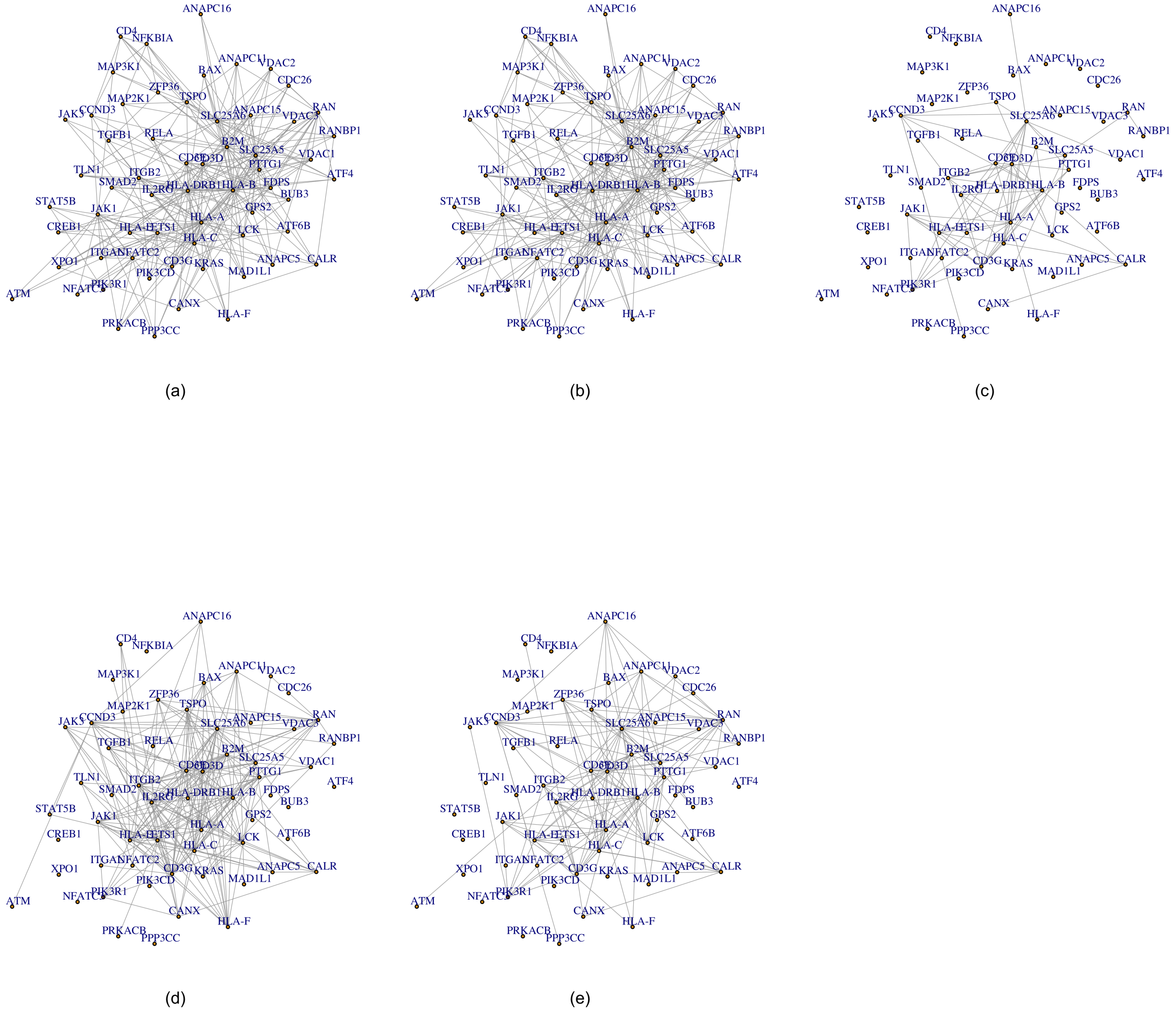

Figure S12: The network structure for 5 subgroups. (a)-(e): the network structures of $1^{(5)}$, $2^{(5)}$, $3^{(5)}$, $4^{(5)}$, and $5^{(5)}$, respectively.

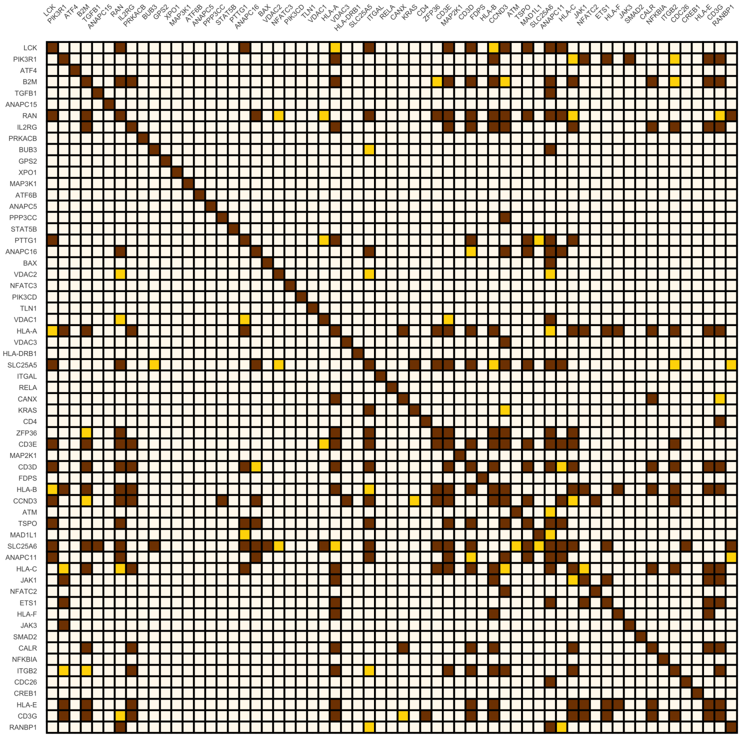

Figure S13: The heatmap illustrating the differences in edges between $5^{(5)}$ and $3^{(3)}$.

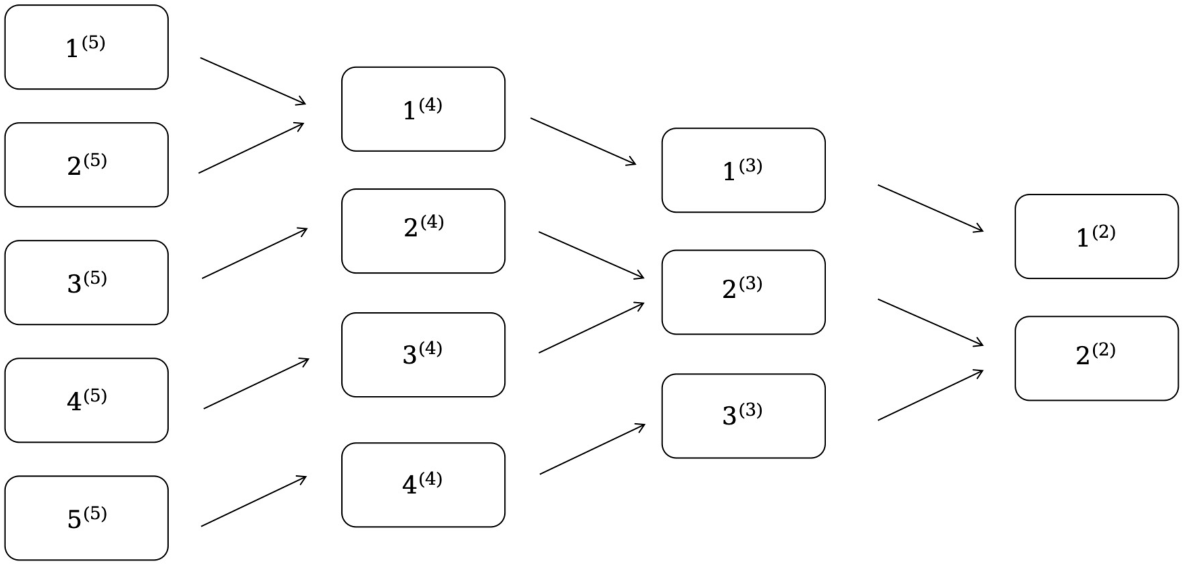

Figure S14: The merged trajectories of the subgroups corresponding to different L.

References

Battini, R., Ferrari, S., Kaczmarek, L., Calabretta, B., Chen, S.-t., and Baserga, R. (1987), "Molecular cloning of a cDNA for a human ADP/ATP carrier which is growth-regulated." *Journal of Biological Chemistry*, 262, 4355–4359.

Bouaziz, J.-D., Ortonne, N., Giustiniani, J., Schiavon, V., Huet, D., Bagot, M., and Bensussan, A. (2005), "Circulating natural killer lymphocytes are potential cytotoxic effectors against autologous malignant cells in sezary syndrome patients," *Journal of investigative dermatology*, 125, 1273–1278.

Chang, Y.-T., Prompsy, P., Kimeswenger, S., Tsai, Y.-C., Ignatova, D., Pavlova, O., Iselin, C., French, L. E., Levesque, M. P., Kuonen, F., et al. (2024), "MHC-I upregulation safeguards neoplastic T cells in the skin against NK cell-mediated eradication in mycosis fungoides," *Nature communications*, 15, 752.

Chevrollier, A., Loiseau, D., Reynier, P., and Stepien, G. (2011), "Adenine nucleotide translocase 2 is a key mitochondrial protein in cancer metabolism," *Biochimica et Biophysica Acta (BBA)-Bioenergetics*, 1807, 562–567.

Danaher, P., Wang, P., and Witten, D. M. (2014), "The joint graphical lasso for inverse covariance estimation across multiple classes," *Journal of the Royal Statistical Society Series B: Statistical Methodology*, 76, 373–397.

Ferenczi, K., Fuhlbrigge, R. C., Kupper, T. S., Pinkus, J. L., and Pinkus, G. S. (2002), "Increased CCR4 expression in cutaneous T cell lymphoma," *Journal of Investigative Dermatology*, 119, 1405–1410.

Gao, C., Zhu, Y., Shen, X., and Pan, W. (2016), "Estimation of multiple networks in gaussian mixture models," *Electronic Journal of Statistics*, 10, 1133–1154.

Hahne, J. C., Okuducu, A. F., Kaminski, A., Florin, A., Soncin, F., and Wernert, N. (2005), "Ets-1 expression promotes epithelial cell transformation by inducing migration, invasion and anchorage-independent growth," *Oncogene*, 24, 5384–5388.

Hanahan, D. and Weinberg, R. A. (2000), "The hallmarks of cancer," *cell*, 100, 57–70.

Hao, B., Sun, W. W., Liu, Y., and Cheng, G. (2018), "Simultaneous clustering and estimation of heterogeneous graphical models," *Journal of Machine Learning Research*, 18, 7981–8038.

He, Y., Li, Q., Hu, Q., and Liu, L. (2022), "Transfer learning in high-dimensional semi-parametric graphical models with application to brain connectivity analysis," *Statistics in medicine*, 41, 4112–4129.

Holterman, C. E., Franovic, A., Payette, J., and Lee, S. (2010), "ETS-1 oncogenic activity mediated by transforming growth factor α," *Cancer research*, 70, 730–740.

Huang, Y., Su, M.-W., Jiang, X., and Zhou, Y. (2015), "Evidence of an oncogenic role of aberrant TOX activation in cutaneous T-cell lymphoma," *Blood, The Journal of the American Society of Hematology*, 125, 1435–1443.

Lanzavecchia, A., Iezzi, G., and Viola, A. (1999), "From TCR engagement to T cell activation: a kinetic view of T cell behavior," *Cell*, 96, 1–4.

Li, S., Cai, T. T., and Li, H. (2022b), "Transfer learning in large-scale Gaussian graphical models with false discovery rate control," *Journal of the American Statistical Association*, 1–13.

Litvinov, I. V., Shtreis, A., Kobayashi, K., Glassman, S., Tsang, M., Woetmann, A., Sasseville, D., Ødum, N., and Duvic, M. (2016), "Investigating potential exogenous tumor

initiating and promoting factors for Cutaneous T-Cell Lymphomas (CTCL), a rare skin malignancy," *Oncoimmunology*, 5, e1175799.

Pan, W. and Shen, X. (2007), "Penalized model-based clustering with application to variable selection," *Journal of Machine Learning Research*, 8, 1145–1164.

Ren, M., Zhang, S., Zhang, Q., and Ma, S. (2022), "Gaussian graphical model-based heterogeneity analysis via penalized fusion," *Biometrics*, 78, 524–535.

Ren, M., Zhen, Y., and Wang, J. (2024), "Transfer learning for tensor Gaussian graphical models," *Journal of Machine Learning Research*, 25, 1–40.

Samelson, L. E. (2002), "Signal transduction mediated by the T cell antigen receptor: the role of adapter proteins," *Annual review of immunology*, 20, 371–394.

Seth, A. and Watson, D. K. (2005), "ETS transcription factors and their emerging roles in human cancer," *European journal of cancer*, 41, 2462–2478.

Tian, Y. and Feng, Y. (2022), "Transfer learning under high-dimensional generalized linear models," *Journal of the American Statistical Association*, 1–30.

Tian, Y., Weng, H., and Feng, Y. (2022), "Unsupervised Multi-task and Transfer Learning on Gaussian Mixture Models," *arXiv:2209.15224*, 1–46.

Wang, B., Zhang, Y., Sun, W. W., and Fang, Y. (2018), "Sparse convex clustering," *Journal of Computational and Graphical Statistics*, 27, 393–403.

Woetmann, A., Lovato, P., Eriksen, K. W., Krejsgaard, T., Labuda, T., Zhang, Q., Mathiesen, A.-M., Geisler, C., Svejgaard, A., Wasik, M. A., et al. (2007), "Nonmalignant T cells stimulate growth of T-cell lymphoma cells in the presence of bacterial toxins," *Blood*, 109, 3325–3332.

Zhou, H., Pan, W., and Shen, X. (2009), "Penalized model-based clustering with unconstrained covariance matrices," *Electronic Journal of Statistics*, 3, 1473–1496.
